# Inside daisies’ evolutionary tangle: new insights into the hybridogenic genus *Anacyclus* (Asteraceae, Anthemideae)

**DOI:** 10.7717/peerj.21638

**Published:** 2026-08-27

**Authors:** Iván Pérez-Lorenzo, Jaume Pellicer, Inés Álvarez, Manica Balant, Lin Fu, Teresa Garnatje, Ilia J. Leitch, Joan Vallès, Daniel Vitales, Oriane Hidalgo

**Affiliations:** 1 Institut Botànic de Barcelona (IBB, CSIC-CMCNB), Barcelona, Spain; 2 Laboratori de Botànica (UB), Unitat Associada al CSIC, Facultat de Farmàcia i Ciències de l’Alimentació—Institut de Recerca de la Biodiversitat (IRBio), Universitat de Barcelona, Barcelona, Catalonia, Spain; 3 Royal Botanic Gardens, Kew, Richmond, United Kingdom; 4 Department of Biodiversity and Conservation, Real Jardín Botánico (CSIC), Madrid, Spain; 5 South China Botanical Garden, Chinese Academy of Sciences, Guangzhou, China; 6 Jardí Botànic Marimurtra—Fundació Carl Faust, Blanes, Spain; 7 Secció de Ciències Biològiques, Institut d’Estudis Catalans, Barcelona, Catalonia, Spain

**Keywords:** *Anacyclus*, Hybridisation, Genome size, Genetic clusters, Plastome, Capitulum morphological characterisation, Mediterranean Basin

## Abstract

**Background:**

The genus *Anacyclus*, distributed across the western Mediterranean Basin, has gained attention as a model system for studying reproductive biology and evolutionary genomics. The genus displays a remarkable diversity in capitulum morphologies, ranging from rayed to rayless forms, while also exhibiting substantial variation in genome size within a context of extensive homoploid hybridisation. However, previous studies are based on fragmented sampling, with the northern Iberian Peninsula underrepresented, and often rely on single individuals per species for genome size estimates.

**Methods:**

To generate a more comprehensive understanding of evolutionary patterns in *Anacyclus*, nuclear ITS and plastid *rpL32-trnL* and *ycf1-rps15* sequences were analysed from 80 individuals representing 47 populations across the distribution range of the genus. Phylogenetic relationships were also reconstructed using complete plastomes and 35S rDNA sequences. Nuclear DNA content was estimated by flow cytometry in 258 individuals from 45 populations. Capitulum morphology was characterised in 75 individuals of *A. clavatus*, *A. valentinus*, and mixed populations. Sampling encompassed all eight recognised species, with particular emphasis on populations from northern Spain.

**Results:**

Re-examination of *Anacyclus* specimens previously identified as *A. homogamos* clarified the debated presence of this species in Catalonia (northeastern Spain), showing that these individuals belong to *A. valentinus*. Nuclear genetic clusters largely correspond to species or species’ groups, although they fail to resolve intraspecific structure and allow only limited detection of hybrids. Genome size analyses confirmed substantial variation of 2C-values across the genus and identified two groups within *A. clavatus* that parallel genetic groups previously identified based on microsatellite data, although a direct correspondence cannot yet be confirmed. Phylogenetic reconstructions based on plastome and nuclear data were strongly discordant, likely reflecting extensive interspecific hybridisation.

**Conclusions:**

Our findings underscore the complexity of *Anacyclus* evolution and emphasise the importance of integrated, population-level studies that combine genomic and phenotypic approaches to better elucidate evolutionary processes in hybridogenic plant lineages.

## Introduction

**Figure 1:**
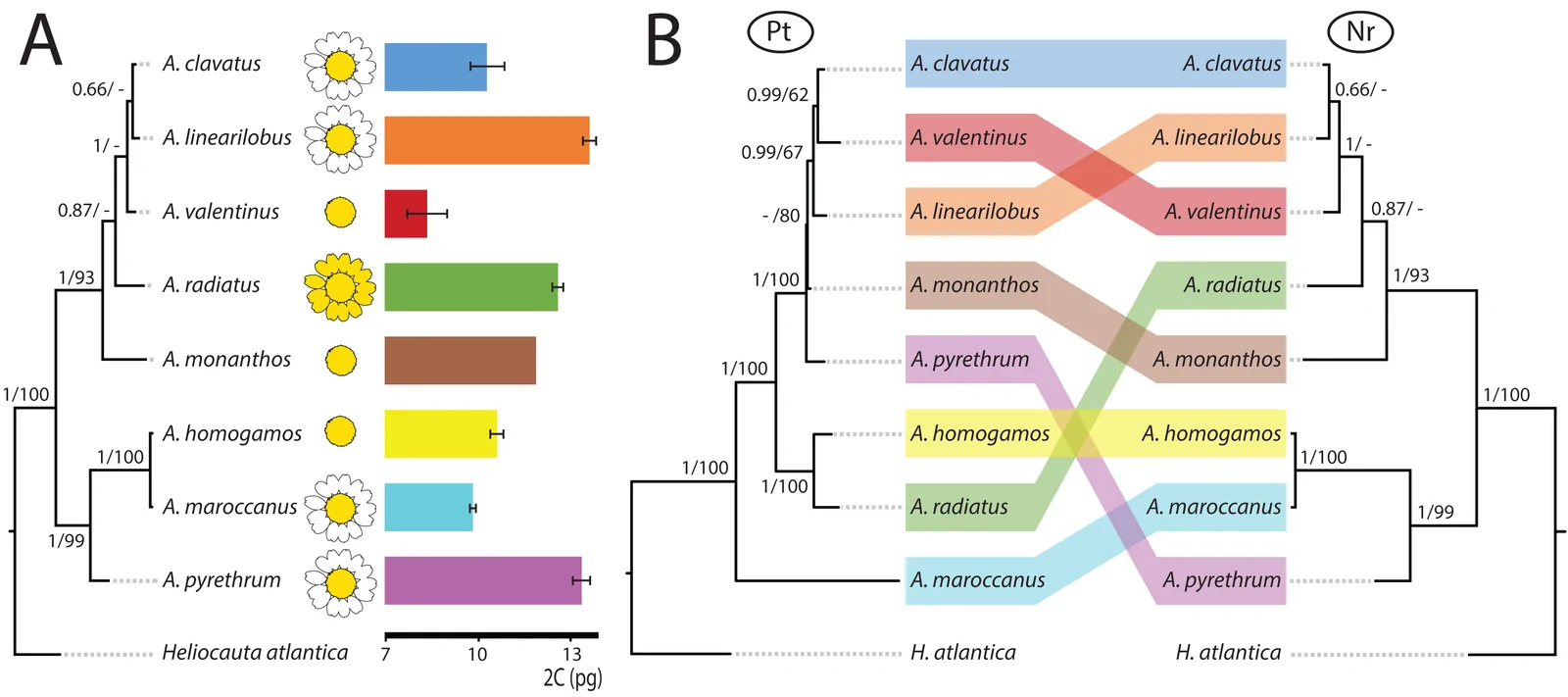
(A) Phylogeny of *Anacyclus* based on the Bayesian analysis of the 35S rDNA region, with indication of capitulum phenotype (ray/rayless and floret colour) alongside bar plots representing the mean genome size value (pg/2C) of each species. (B) Tanglegram of phylogenies inferred from Bayesian analysis of the plastomes (Pt) (left) and of the nuclear (Nr) 35S rDNA region (right). Posterior probabilities and bootstrap support values from the maximum likelihood analysis are indicated on the branches.

Hybridising lineages serve as especially valuable models for investigating the evolution of phenotypic traits, as well as the mechanisms driving reproductive isolation and speciation (Thompson et al., 2024). This holds particularly true when the taxa involved display contrasting morphologies that influence reproductive fitness, such as the genus *Anacyclus*, whose species exhibit either rayed or rayless capitula (Fig. 1A; Humphries, 1979; Cerca et al., 2019). The presence of ray florets is considered an important trait for reproductive success in Asteraceae and is markedly labile in the Anthemideae tribe, where at least 20 genera, including *Anacyclus*, present both rayed and rayless capitula (Bello et al., 2017). *Anacyclus* species are considered to be interfertile (Humphries, 1981) and predominantly self-incompatible—at least in the few species for which the breeding system has been studied (self-incompatibility reported in *Anacyclus clavatus*, *A. homogamos*, and *A. valentinus*; Álvarez et al., 2020; mixed mating system in *A. linearilobus*, Sánchez-Albert et al., 2022). Among the numerous hybrids documented within the genus, those between *A. clavatus* (with patent white ligulate florets) and *A. valentinus* (with small, inconspicuous yellow ligulate florets) are particularly noteworthy, as they display a wide range of intermediate phenotypes (Bello et al., 2013; Lozano, 2016). As a result, their contact zones are attracting increased attention, being considered natural laboratories (Agudo et al., 2019; Cerca et al., 2019; Álvarez et al., 2020; Agudo et al., 2023). Furthermore, individuals presenting capitula with peripheral ‘trumpet’ peloric florets (*i.e.,* actinomorphic florets replacing the normally zygomorphic ones) are sporadically found in natural populations of both species and are also becoming a focus of research (Bello et al., 2013; Bello et al., 2017). Such findings have, in recent years, led to a stronger focus on understanding the phylogenetic and genomic backgrounds of the genus, a knowledge essential for reconstructing the evolutionary trajectories of reproductive traits.

Molecular phylogenetic tools have redefined the circumscription of *Anacyclus* (Vitales et al., 2018), reducing the genus from eleven species to eight after three former species were transferred to the genus *Cota* (*i.e., C. anatolica*, *C. latealata* and *C. nigellifolia*; Vitales et al., 2018). The most recent phylogenies of *Anacyclus*, though still not yet fully resolved, nevertheless provide support for two main clades (Oberprieler, 2004; Vitales et al., 2018). The first clade includes the North African *A. linearilobus*, an endemic species with a very restricted range, together with *A. clavatus*, *A. radiatus*, and *A. valentinus*, species with broad distribution ranges spanning North Africa, parts of Spain, and other regions of the Mediterranean Basin. The second clade comprises the North African species *A. homogamos* and *A. maroccanus*, as well as *A. pyrethrum*, which is primarily North African but has some populations extending into southern Spain. It has been suggested that *A. homogamos* might also have a few populations in Spain, particularly in Catalonia, in the northeast of the country (Molero & Pyke, 2019 and references therein), although these records remain doubtful (Agudo et al., 2019; Álvarez, 2019). Finally, *A. monanthos*, a North African species, occupies either an unresolved position in a trichotomy with the two clades (Vitales et al., 2018), or is closely related to *A. clavatus* (Oberprieler, 2004) or *A. radiatus* (Vitales et al., 2020). Species with rayed and rayless capitula appear scattered across the phylogeny, suggesting multiple events of parallel evolution for this trait (Fig. 1A).

**Figure 2:**
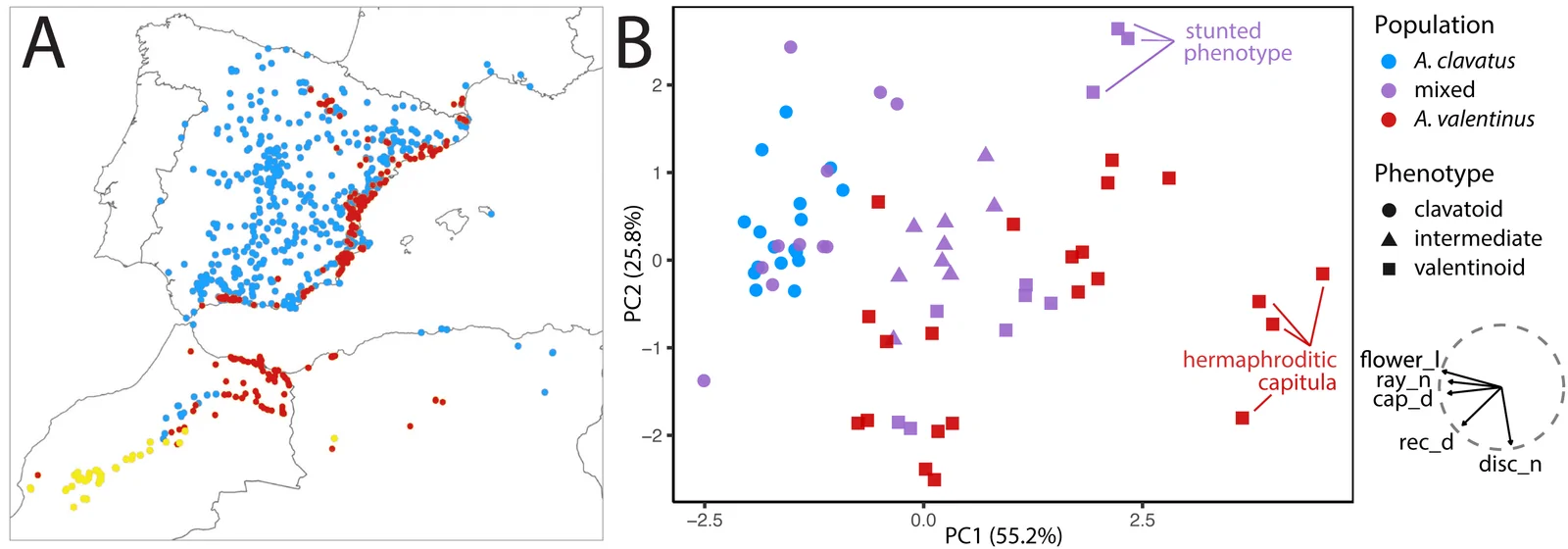
(A) Geographic distribution of *Anacyclus clavatus* (blue dots), *A. homogamos* (yellow dots), and *A. valentinus* (red dots), displayed on a map modified from Agudo et al. (2023). (B) Principal component analysis (PCA) showing the distribution of capitulum traits in *A. clavatus*, *A. valentinus* and mixed populations. Flower_l: length of the ligulate flowers, ray_n: number of ligulate flowers, cap_d: diameter of the capitulum, rec_d: diameter of the receptacle, disc_n: number of tubular flowers. A PCA correlation circle is provided.

Hybridisation, and more specifically homoploid hybridisation, is considered a major evolutionary driver within the genus. It is readily detectable in current hybrid zones through the phenotype, genome size, and population genetics (Bello et al., 2013; Lozano, 2016; Agudo et al., 2019; Agudo et al., 2023), and its signature has also been inferred at the genus level from distinct molecular patterns in the genome. In particular, homoploid hybridisation has been suggested to explain the hypervariability of the 35S ribosomal DNA (rDNA) sites (Rosato et al., 2017) and the presence of interstitial telomeric-like repeats on the chromosomes (Rosato et al., 2018). Homoploid hybridisation also likely accounts for the variation in genome size across *Anacyclus* species, ranging from 7.70 pg/2C in *A. valentinus* to 13.14 pg/2C in its sister species, *A. linearilobus* (Agudo et al., 2019; Vitales et al., 2020). Indeed, a study of the repeatome has shown that such C-value variation is not actually associated with the activation of transposable elements, but rather it arises from the exchange of genetic material through hybridisation and *via* balanced rearrangements involving chromosomal segment exchanges (Vitales et al., 2020). Genome size data are nevertheless still mostly limited to a single individual measured per species (Vitales et al., 2020). The only exceptions are *A. clavatus*, *A. homogamos* and *A. valentinus* where genome size variation has been studied at the population level (Agudo et al., 2019), albeit with sparse representation of their northeastern distribution in Spain, where the species also coexist (Fig. 2A).

In this study, we address several unresolved questions concerning the diversity and evolution of *Anacyclus*, building on previous work and available material, and integrating newly generated data to fill remaining knowledge gaps. Specifically, we (i) examine the debated presence of *A. homogamos* in Catalonia, (ii) reassess phylogenetic relationships and population genetic structure within the genus using nuclear and plastid DNA sequences, and (iii) expand the available genome size data and analyse patterns of genome size variation across the genus and within the *A. clavatus*–*A. valentinus* hybrid complex. To this end, we sampled all eight currently recognised species of *Anacyclus*, with particular emphasis on the northern part of the genus distribution in Spain, where we also conducted a detailed morphological characterisation of capitulum traits. Together, these approaches contribute to a more complete understanding of the genus and provide a broader framework for future evolutionary studies.

## Materials & Methods

### Sampling

The *Anacyclus* material deposited in the Botanical Institute of Barcelona (BC) and University of Barcelona (BCN) herbaria was studied to search for specimens identified as *A. homogamos*. We particularly sought the specimens which had been used as evidence for the proposed presence of *A. homogamos* in Catalonia, in the provinces of Barcelona, Girona and Tarragona (Molero & Pyke, 2019). We also surveyed the natural populations growing around Barcelona to collect samples exhibiting a phenotype similar to these putative “*A. homogamos*” specimens. Morphological characterisation of capitula was limited to those samples, along with populations from the northern distribution of *A. clavatus* and *A. valentinus*. Dissections and herbarium vouchers from these collections are deposited in the BC herbarium and collection details are provided in Table S1.

Genome size was estimated for 258 individuals from 45 populations including (i) individuals clearly identified as *A. clavatus* (eight), *A. homogamos* (three), *A. linearilobus* (four), *A. maroccanus* (three), *A. monanthos* (two), *A. pyrethrum* (two), *A. radiatus* subsp. *coronatus* (one), *A. radiatus* subsp. *radiatus* (three), *A. valentinus* (six); (ii) individuals from mixed populations of *A. clavatus* and *A. homogamos* (one), *A. clavatus* and *A. radiatus* (one), *A. clavatus* and *A. valentinus* (eight), *A. homogamos* and *A. valentinus* (one); (iii) individuals from populations of *Cota nigellifolia* (two). Collection details are provided in Table S2.

Nuclear and plastid sequencing was performed on 80 and 75 individuals, respectively, from 47 *Anacyclus* populations including (i) individuals clearly identified as *A. clavatus* (11), *A. homogamos* (three), *A. linearilobus* (two), *A. maroccanus* (three), *A. monanthos* (three), *A. pyrethrum* (two), *A. radiatus* (six), *A. valentinus* (13), and (ii) individuals collected from mixed populations of *A. clavatus* and *A. valentinus* (four). Individuals from two populations of *Heliocauta atlantica* were also sequenced and used as the outgroup, as the genus has been shown to be sister to *Anacyclus* in recent phylogenetic reconstructions of Anthemideae (Vitales et al., 2018; Criado-Ruiz et al., 2025). Herbarium vouchers are deposited in the BC and Real Jardín Botánico (MA) herbaria; collection details are provided in Table S3.

### Phenotypic characterisation

Morphological characterisation followed the protocol of Fu et al. (2023). Measurements and counts of florets were performed on the dissection scans using Fiji ImageJ v. 2.9.0 (Schindelin et al., 2012). Six capitulum traits were considered: capitulum diameter, receptacle diameter, length and width of ligulate florets, number of ligulate florets and number of tubular florets. Florets showing signs of folding or damage were excluded from the measurements. Data analysis and visualisation were performed in R v. 4.4.2 (R Core Team, 2024) using the ggplot2 package (Wickham et al., 2019). Pairwise differences among groups were tested using Wilcoxon rank-sum tests with Benjamini–Hochberg correction applied to adjust *p*-values for multiple comparisons. Trait associations were evaluated using Spearman’s rank correlations, calculated both across all individuals and separately within each group. Prior to principal component analysis (PCA), limb width of ligulate florets was excluded to ensure a correlation threshold of *r* < 0.950 among variables (Fig. S1). The remaining capitulum traits were log1p-transformed to accommodate zero values and reduce skewness and then standardized by centering to a mean of zero and scaling to unit variance (standard deviation = 1). PCA was conducted using the prcomp function.

### Flow cytometry measurements

Nuclear DNA contents were estimated using the flow cytometers CyFlowSL and CyFlow Space (Sysmex-Partec, Norderstedt, Germany) fitted with a 100-mW green solid-state laser (Cobolt Samba), following the one-step protocol of Doležel, Greilhuber & Suda (2007) with minor modifications as described by Clark et al. (2016). Measurements were made with the internal standards *Petroselinum crispum* ‘Champion Moss Curled’ (4.5 pg/2C; Obermayer et al., 2002) for *Anacyclus* species, and *Pisum sativum* ‘Ctirad’ (9.09 pg/2C; Doležel et al., 1998) for individuals from a population of *Cota nigellifolia*. The ‘general purpose buffer’ (GPB; Loureiro et al., 2007) was used, supplemented with 3% polyvinylpyrrolidone (PVP-40). Two replicates of each individual were measured. The nuclear DNA content of each sample run was estimated by recording at least 1,000 nuclei per fluorescence peak. Resulting output histograms were analysed using the FlowMax software (v. 2.9, Sysmex-Partec GmbH) for statistical calculations. Genome size data from a previously conducted population-level study on *Anacyclus* (Agudo et al., 2019) were incorporated into the subsequent analyses. Additionally, genome size estimates from Vitales et al. (2020)—which, as noted earlier, were limited to a single individual per species—were also included and complemented by new data obtained from multiple individuals within the same populations.

### DNA sequencing and analysis

Approximately 20 mg of leaf tissue preserved in silica gel were used for DNA extraction using the CTAB (Doyle & Doyle, 1987) protocol with minor modifications. The quality of the DNA extraction was analysed using a NanoDrop 1,000 spectrophotometer (ThermoScientific, Wilmington, DE, USA). The nuclear ITS region and the intergenic regions of the plastid *rpL32-trnL* and *ycf1-rps15* were amplified following the procedure described in Vitales et al. (2018), and sequenced with Big Dye Terminator Cycle Sequencing v. 3.1 (PE Biosystems, Foster City, CA, USA) at the Genomics Laboratory of the Scientific and Technological Centers of the University of Barcelona (CCiTUB) on an ABI PRISM 3700 DNA analyzer (PE Biosystems). The three intergenic regions studied were assembled using BioEdit v. 7.1.3.0 (Hall, 1999), aligned with ClustalW Multiple Alignment v. 1.4 (Thompson, Higgins & Gibson, 1994) and adjusted manually. GenBank accession numbers are provided in Table S3. For the analysis of the ITS region, a NeighborNet network (Bryant & Moulton, 2004) was computed using SPLITSTREE4 (Huson, 1998), transforming sequence divergences to uncorrected P-distances and handling ambiguous IUPAC characters (derived from polymorphisms detected in electropherograms) as average states. The statistical support of the clades was obtained with a bootstrapping of 1,000 pseudoreplicates and forming groups with a support value ≥ 85%. To illustrate the relationships between the different plastid haplotypes obtained from the intergenic spacers *rpL32-trnL* and *ycf1-rps15*, a parsimony network was computed using TCS within PopArt software (Leigh & Bryant, 2015), with a 95% confidence interval for the maximum number of differences for a change in sequence between haplotypes.

### Plastome and 35S rDNA assemblies and phylogenetic analyses

Plastomes of *Anacyclus* species and *Heliocauta atlantica* were assembled from previously published Illumina sequencing data deposited in the NCBI short-read archive (SRA) under BioProject accession number PRJNA556380. Plastid genome assemblies were done using NOVOPlasty v. 4.2.1 (Dierckxsens, Mardulyn & Smits, 2017) with default parameters after removing the adapters (using the fastp pipeline; Chen et al., 2018), as recommended by the authors. The *rbcL* gene of *A. valentinus* (NCBI GenBank: GU817737) was used as seed of the analysis and, if the resulting assembly was not circularized, an alternative seed sequence was employed (*i.e.,* the *psbA-trnH* region, NCBI GenBank: GU818332). The resulting contigs were aligned to the published *Achillea millefolium* plastome (NCBI GenBank: ON320384) using MAFFT (Katoh & Standley, 2013), and the alignment was subsequently edited with Geneious Prime v. 2023.2.1 (Kearse et al., 2012) to ensure consistent orientation of short single copy and inverted repeat regions across all sequences. Filtered Illumina reads from each species were then mapped to the corresponding reconstructed plastome to obtain consensus sequences, following the procedures described by Pascual-Díaz et al. (2025). Each consensus plastome was annotated using GeSeq (Tillich et al., 2017) available through the platform MPI-MP CHLOROBOX (https://chlorobox.mpimp-golm.mpg.de/, accessed 11 February 2024). Subsequently, all automatically annotated consensuses were checked manually using Geneious. The annotated plastid genomes were submitted to GenBank.

The reconstructed plastomes of *Anacyclus* and *H. atlantica*, together with the published plastid genome of *Achillea millefolium* (NCBI GenBank: ON320384), were used for phylogenetic inference. One copy of the inverted repeat of all plastomes was manually removed in all analyses to avoid data duplication, subsequently partitioning the sequences based on the annotation of CDS, tRNA, rRNA and non-coding regions. The resulting sequences were aligned using MAFFT (Katoh & Standley, 2013). PartitionFinder2 (Lanfear et al., 2017) was used to fit the best nucleotide substitution model for all the different partitions. Bayesian inference (BI) phylogenetic analysis was conducted using MrBayes v. 3.2.6 (Ronquist et al., 2012). Markov Chain Monte Carlo (MCMC) with two independent chains were run for 1,000,000 generations and trees sampled every 1,000 generations. Convergence was assessed by verifying that the average standard deviation of split frequencies was below 0.01 and that the potential scale reduction factor was near 1.0 for all parameters. The first 25% of the trees were discarded as “burn-in”, and the posterior probability was estimated by constructing the 50% majority-rule consensus tree. Maximum likelihood (ML) analyses were conducted using the program RAxML v. 8.2.10 (Stamatakis, 2014) with 1,000 bootstrap replicates, the GTR+G substitution model for all partitions and other parameters using the default settings. The phylogenetic trees were visualised with FigTree v. 1.4.5 (Rambaut, 2024), using as outgroup *Achillea millefolium*.

To validate the topology obtained with whole plastome data (*i.e.,* CDS, tRNA, rRNA and non-coding regions) an additional phylogenetic reconstruction was performed only using the CDS regions. Only the BI approach was used to perform the phylogenetic analysis for this dataset, employing the same parameter selection options as mentioned above. The consensus sequences of the 35S rDNA unit for each species were obtained from the same Illumina sequencing data (PRJNA556380), following a combined approach involving *de novo* and reference-guided assembly methodology, as explained in Vitales et al. (2020). For the outgroup species *A. millefolium*, Illumina sequences were obtained from NCBI short-read archive (SRR17032110) and the consensus sequence of 35S rDNA unit was obtained using the same methodology (NCBI Genbank: PZ437183). Due to the presence of multiple subrepeats, the 3′ETS region was difficult to align and therefore excluded from further analyses. The annotated 35S rDNA sequences were then submitted to GenBank (Table S4). The same phylogenetic approaches employed for plastome analyses, but in this case partitioning the sequences by spacer (5′ETS, ITS1 and ITS2) and genic regions (18S, 5.8S and 26S), were used for the phylogenetic inference based on 35S rDNA data.

**Figure 3:**
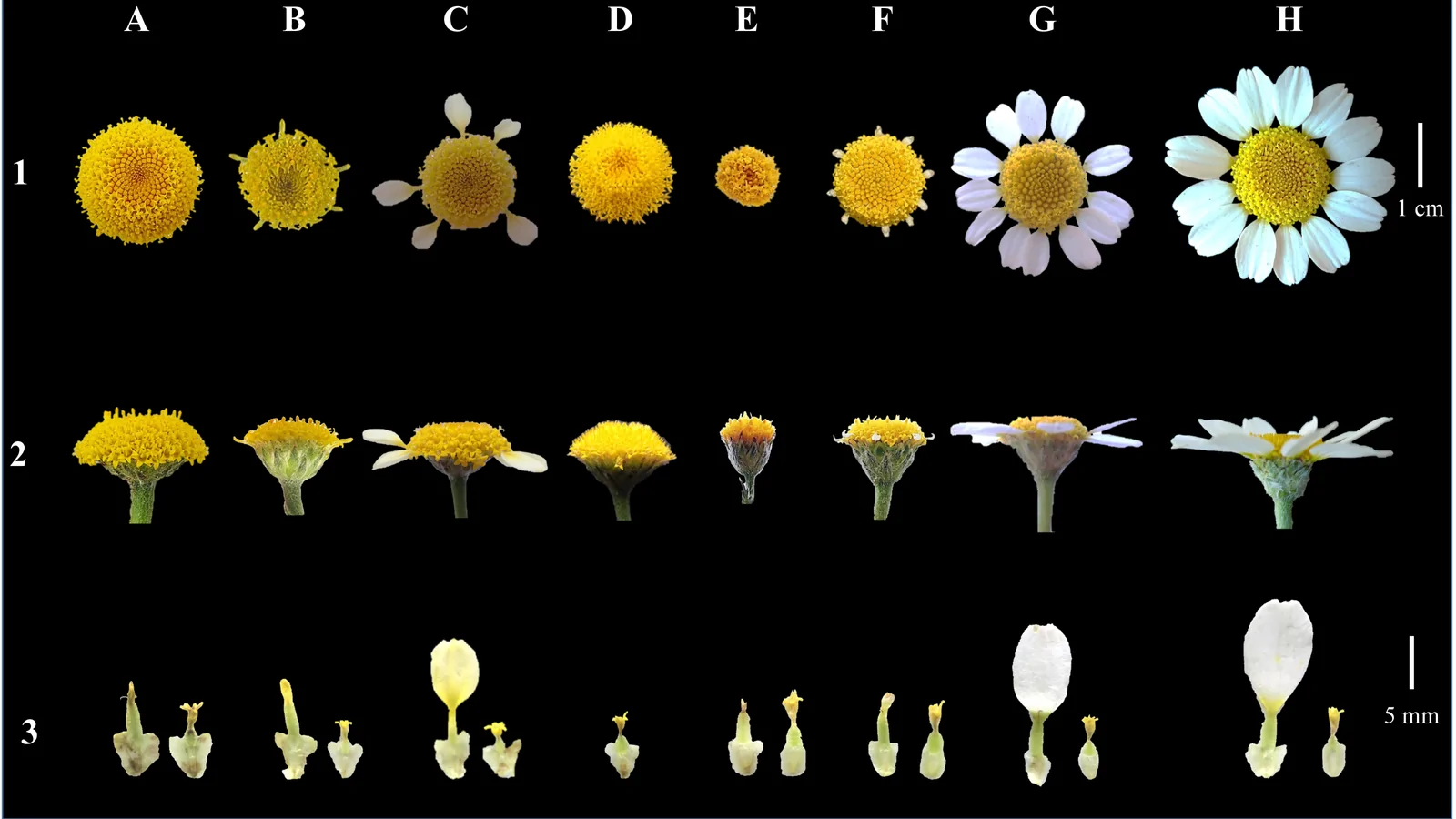
Capitula of *Anacyclus valentinus* (A–D), mixed (E–G) and *A. clavatus* (H) populations. Frontal (1) and lateral (2) views, and detail of a ligulate and a tubular floret (3). Corresponding populations: MON (A, C), CAL (B, D), COL (E–F), SBO (G), BOT (H).

## Results

### Morphological characterisation

Examination of *Anacyclus* specimens deposited in the BC and BCN herbaria resulted in the retrieval of three specimens determined as *A. homogamos* (Fig. S2). Two of them, BCN-31856 and BCN-125055, were cited in Molero & Pyke (2019). The third, BC-978956, was collected in 2019 in Montjuïc, Barcelona. Close examination of the capitula of these specimens revealed the presence of inconspicuous ligulate florets in each case (Fig. S2). In addition, we sampled a population (COL) near Barcelona with individuals displaying a similar phenotype as those of the aforementioned herbarium specimens (*i.e.,* small capitula with no visible ligulate florets and a reduced number of tubular florets, resembling *A. homogamos* or stunted *A. valentinus* capitula). This population was phenotypically heterogeneous (hereafter referred as mixed). It included individuals with a morphology similar to those specimens previously identified as *A. homogamos* (valentinoid phenotype; Fig. 3E), individuals with a morphology resembling *A. clavatus* (clavatoid phenotype), as well as individuals with an intermediate morphology between *A. clavatus* and *A. valentinus* (intermediate phenotype). Results from the morphological characterisation of the capitula in this population (*i.e.,* COL), together with capitula analysed from other selected *A. clavatus*, *A. valentinus*, and mixed populations are presented in Table 1 (mean values for populations), Table S1 (raw data) and Fig. S1. PCAs of capitulum traits are shown in Fig. 2B (morphospaces occupied by *A. clavatus*, *A. valentinus* and mixed populations), Fig. S3A (morphospaces occupied by *A. clavatus*, *A. valentinus* and mixed populations with individual identification), and Fig. S3B (morphospaces occupied by each population with individual identification).

**Table 1:** Morphological characterisation of the capitula of *Anacyclus clavatus*, *A. valentinus* and mixed populations. Raw data are presented in Table S1.

**Accession**			**Phenotype**	**Capitulum**		**Ray florets**				**Disc florets**
**Population**	**Code**	**N Ind.**	**(mixed pop.)**	*ϕ* (SD)^*^	Receptacle *ϕ* (SD)^*^	**N (SD)**	Flower length (SD)^*^	Limb width (SD)^*^	**Colour**	**N (SD)**
*Anacyclus clavatus*	BOT	1		32.11	4.72	13	12.13 (0.27)	5.65 (0.19)	white	252
	STG	10		35.32 (3.98)	4.12 (0.46)	12.3 (1.16)	13.33 (1.05)	5.75 (0.57)	white	207.8 (36.81)
	JAC	6		33.90 (2.37)	4.93 (0.65)	13.33 (1.33)	12.9 (1.1)	6.11 (0.95)	white	187.83 (36.78)
Mixed	COL	1	clavatoid	29.83	4.34	9	11.21 (0.35)	5.3 (0.23)	white	152
	COL	1	intermediate	15.82	3.31	9	4.56 (0.37)	1.02 (0.06)	white	206
	COL	3	valentinoid	8.89 (1.13)	2.41 (0.23)	7.33 (1.15)	2.95 (0.52)	0.33 (0.1)	yellow	127 (14)
	SBO	10	clavatoid	35.23 (4.24)	4.01 (1.40)	11.60 (1.71)	13.43 (1.66)	5.67 (0.8)	white	216.20 (58.71)
	SBO	10	intermediate	21.12 (2.53)	3.59 (0.54)	10 (1.76)	5.85 (0.65)	1.74 (0.58)	white/ yellow	244.67 (36.49)
	SBO	10	valentinoid	18.89 (1.76)	4.11 (1.36)	10 (2.56)	3.18 (0.48)	0.59 (0.26)	yellow	316.6 (56.36)
*Anacyclus valentinus*	MON	12		24.14 (4.20)	5.08 (0.95)	7 (2.92)	5.99 (2.42)	1.47 (1.13)	yellow	346.33 (79.78)
	CAL	11		16.39 (0.8)	2.74 (0.60)	2.55 (1.97)	3.29 (0.9)	0.64 (0.12)	yellow	247.27 (35.19)

**Notes.**

SD standard deviation

* Measured in mm.

### Genome size data

**Figure 4:**
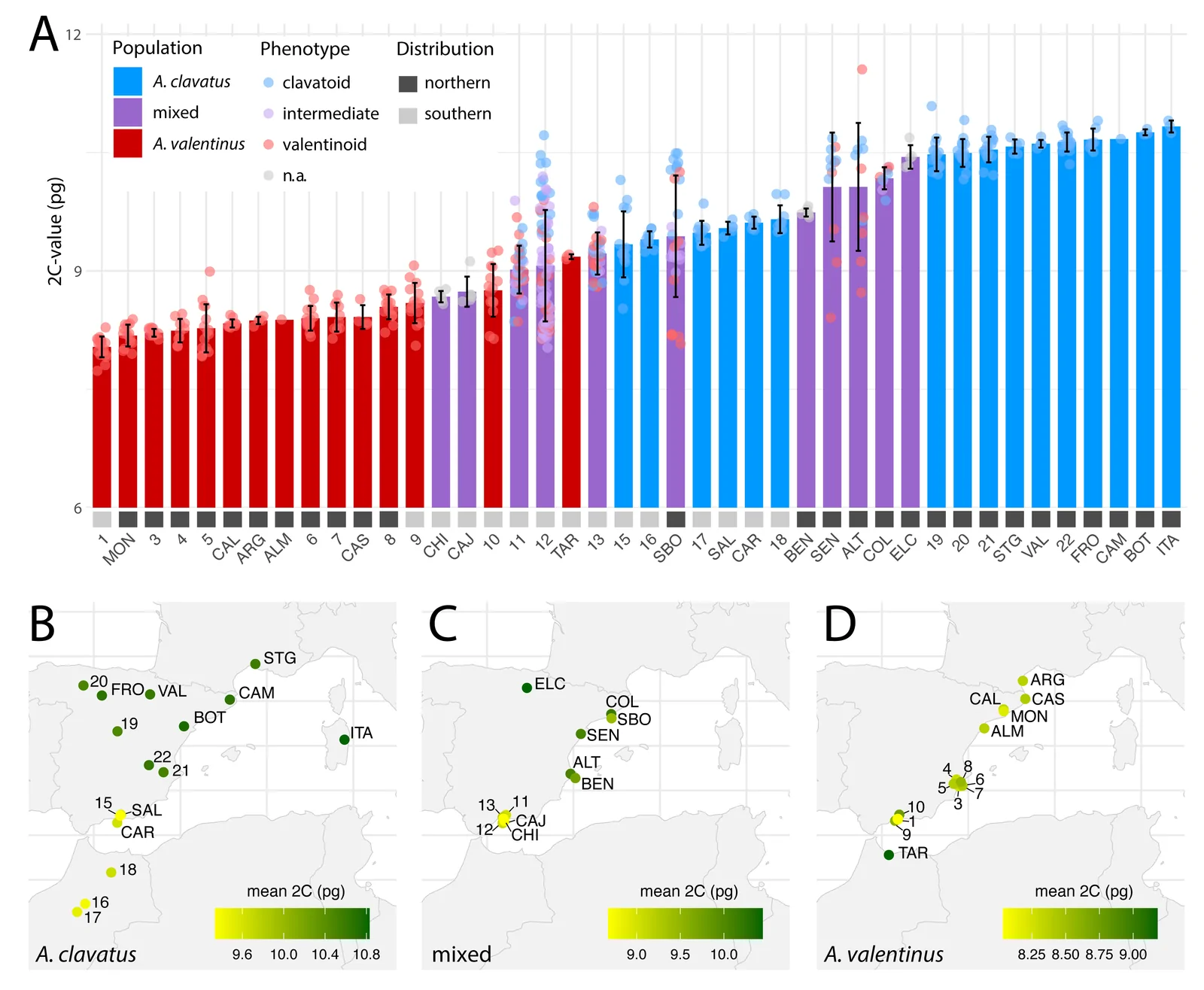
(A) Bar plots showing the mean and standard deviation of 2C-values for *Anacyclus clavatus*, *A. valentinus*, and mixed populations, based on data from this study (Table 2), Agudo et al. (2019) with population codes indicated as numbers, Vitales et al. (2020). Individual data points are overlaid on the bars, with color indicating the phenotype. The grey rectangle above each population code provides a rough indication of geographic origin: light grey for southern populations and dark grey for northern populations, with the northern boundary of Andalusia serving as the approximate dividing line. (B–D) Mapped distribution of mean genome size values of *A. clavatus* (B), mixed (C) and *A. valentinus* populations (D).

Genome size data are presented in Table 2 (mean values for populations and taxa) and Table S2 (values for individuals). Figure 1A shows the mean genome size value of *Anacyclus* species plotted onto the phylogeny of the genus. Figure 4 presents the geographic distribution and diversity of mean genome size values for *Anacyclus valentinus*, *A. clavatus*, and mixed populations, based on data from this study as well as from Agudo et al. (2019) and Vitales et al. (2020). Although mixed populations generally exhibited high variability, the distribution of 2C-values within them revealed contrasting patterns, ranging from gradual transitions (*e.g.*, population 12; Fig. 4A) to distributions centered around distinct values (*e.g.*, population SBO; Fig. 4A). In some cases, the data matched the expected pattern, with valentinoid phenotypes showing the smallest C-values, clavatoid phenotypes the largest, and intermediate values corresponding to intermediate phenotypes. However, numerous exceptions were seen. The most striking was a valentinoid individual (ALT 35-7) from population ALT which had the highest 2C-value in the entire dataset (11.55 pg/2C). Flow cytometry histograms showing the individual measurement for this specimen as well as the combined measurement of this individual with the valentinoid individual that had the smallest genome size within the same population (ALT 35-4), are given in Figs. S4A and S4B respectively.

**Table 2:** Mean genome size values for populations and species. Pop, population; SD, standard deviation; min, lowest 2C value in the population; max, highest 2C value in the population; N, number of individuals assessed. ^1^Phenotypic data are provided for mixed populations when available. Values in bold indicate species/group-level genome size statistics (mean 2C value and SD, minimum and maximum values) and the total number of individuals assessed per species/group. ^2^Mean 2C and standard deviation (SD) across mixed populations were calculated using mean values from individual populations, irrespective of phenotypic variation.

**Species in population**	**Code pop.**	**Phenotype** ^**1**^	**2C (pg)**	**SD**	**Min**	**Max**	**N**
***Anacyclus clavatus***	**BOT**		10.75	0.04	10.73	10.80	3
	**CAM**		10.67	–	10.67	10.67	1
	**CAR**		9.61	0.08	9.52	9.72	7
	**FRO**		10.66	0.14	10.51	10.90	7
	**ITA**		10.83	0.08	10.75	10.90	3
	**SAL**		9.54	0.08	9.43	9.65	5
	**STG**		10.57	0.09	10.48	10.69	5
	**VAL**		10.61	0.05	10.55	10.70	6
			**10.41**	**0.52**	**9.43**	**10.90**	**37**
***Anacyclus clavatus*, *A. homogamos***	**OUA**		9.74	0.08	9.62	9.81	5
			**9.74**	**–**	**9.62**	**9.81**	**5**
***Anacyclus clavatus*, *A. radiatus***	**TORR**		9.78	0.47	9.39	10.60	5
			**9.78**	**–**	**9.39**	**10.60**	**5**
***Anacyclus clavatus, *A. valentinus****	**ALT**	clavatoid	10.37	0.41	9.64	10.65	5
	**ALT**	valentinoid	9.81	1.01	8.73	11.55	6
	**BEN**		9.74	0.05	9.68	9.82	6
	**CAJ**		8.74	0.19	8.61	9.12	6
	**CHI**		8.67	0.07	8.58	8.77	6
	**COL**	clavatoid	10.09	0.18	9.89	10.23	3
	**COL**	intermediate	10.16	0.14	10.03	10.31	3
	**COL**	valentinoid	10.26	0.07	10.19	10.32	3
	**ELC**		10.44	0.15	10.32	10.69	5
	**SBO**	clavatoid	10.20	0.34	9.46	10.49	10
	**SBO**	intermediate	9.23	0.15	8.98	9.56	10
	**SBO**	valentinoid	8.88	0.85	8.08	10.25	10
	**SEN**	clavatoid	10.42	0.18	10.17	10.68	6
	**SEN**	valentinoid	9.71	0.84	8.41	10.57	6
			**9.67^2^**	**0.67^2^**	**8.08**	**11.55**	**85**
***Anacyclus homogamos***	**ASK**		10.6	–	10.6	10.6	1
	**ASN**		10.75	0.11	10.6	10.85	4
	**IMO**		10.39	0.15	10.2	10.55	5
			**10.58**	**0.18**	**10.2**	**10.85**	**10**
***Anacyclus homogamos*, *A. valentinus***	**KSI**		9.91	0.08	9.80	10.00	5
			**9.91**	**–**	**9.8**	**10**	**5**
***Anacyclus linearilobus***	**BMO**		13.41	0.17	13.21	13.65	5
	**BOM**		13.45	0.10	13.30	13.66	12
	**MAC**		13.83	0.12	13.64	13.95	6
	**MOB**		13.43	0.10	13.32	13.52	3
			**13.53**	**0.20**	**13.21**	**13.95**	**26**
***Anacyclus maroccanus***	**MAR**		9.84	0.09	9.69	9.91	5
	**SID**		9.75	0.08	9.65	9.81	4
	**TNI**		9.83	0.01	9.82	9.84	2
			**9.80**	**0.05**	**9.65**	**9.91**	**11**
***Anacyclus monanthos***	**GAB**		11.83	–	11.83	11.83	1
	**MAT**		11.83	–	11.83	11.83	1
			**11.83**	**0**	**11.83**	**11.83**	**2**
***Anacyclus pyrethrum***	**ANS**		13.39	0.05	13.32	13.44	5
	**PEN**		12.71	–	12.71	12.71	1
			**13.05**	**0.48**	**12.71**	**13.44**	**6**
***Anacyclus radiatus*** **subsp.** ***coronatus***	**ESS**		12.57	0.18	12.28	12.72	5
			**12.57**	**–**	**12.28**	**12.72**	**5**
***Anacyclus radiatus*** **subsp.** ***radiatus***	**BUR**		12.59	0.21	12.43	12.94	5
	**CHE**		12.41	0.15	12.22	12.56	4
	**ENT**		12.48	0.12	12.32	12.65	5
			**12.49**	**0.09**	**12.22**	**12.94**	**14**
***Anacyclus valentinus***	**ALM**		8.38	–	8.38	8.38	1
	**ARG**		8.37	0.05	8.30	8.43	5
	**CAL**		8.34	0.05	8.28	8.44	10
	**CAS**		8.42	0.15	8.32	8.64	4
	**MON**		8.18	0.14	7.95	8.39	14
	**TAR**		9.18	0.03	9.15	9.21	3
			**8.48**	**0.35**	**7.95**	**9.21**	**37**
***Cota nigellifolia***	**Ker**		18.37	0.09	18.25	18.46	5
	**101336**		18.81	0.13	18.65	18.96	5
			**18.59**	**0.31**	**18.25**	**18.96**	**10**

### Genetic data

**Figure 5:**
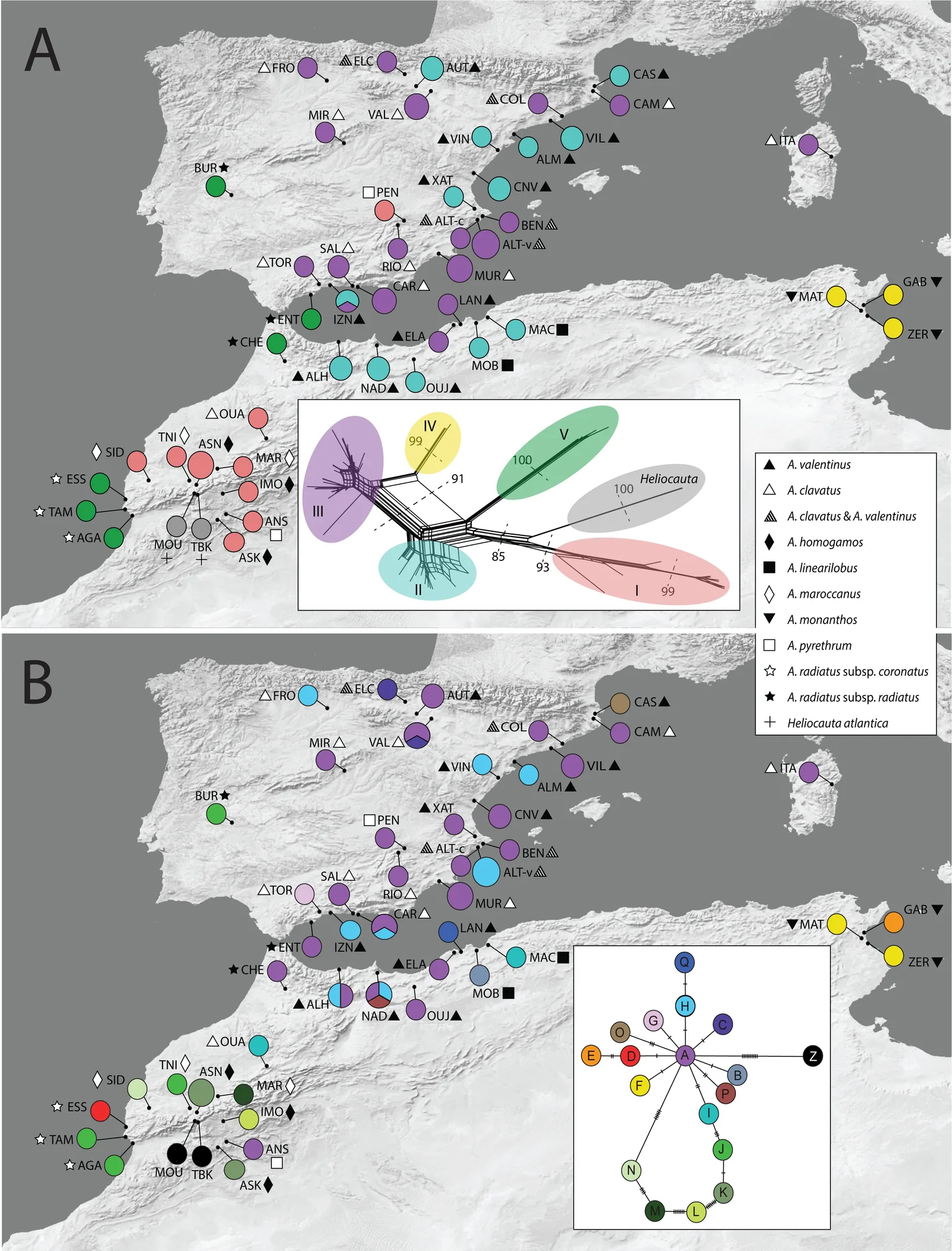
(A) Geographic distribution of nuclear genetic groups and NeighborNet network, resulting from the analysis of the ITS. Bootstrap values equal to or greater than 85% are shown for the genetic groups detected. (B) Geographic distribution of haplotypes found in *Anacyclus* and statistical parsimony network, resulting from the analyses of the *rpL32-trnL* and *ycf1-rps15*. Crossing bars indicate one base mutation distance. The size of the circles in the maps and the network represents the number of individuals.

Based on the analysis of the ITS region, five genetic clusters (I–V) were identified across *Anacyclus* populations (Fig. 5A). Results showed a strong correspondence between species or species’ groups and nuclear genetic clusters. Two species formed their own genetic cluster, *A. monanthos* (cluster IV) and *A. radiatus* (cluster V). Populations of *A. homogamos*, *A. maroccanus*, and *A. pyrethrum* all grouped within cluster I. Nearly all populations of *A. clavatus*, except one (*i.e.,* OUA, in cluster I), were grouped within cluster III. This cluster also included two populations of *A. valentinus* (ELA and LAN), and all mixed *A. clavatus* and *A. valentinus* populations. Cluster II comprised all *A. valentinus* (except ELA and LAN) and all *A. linearilobus* populations. Our results only found one example of intra-population heterogeneity: the IZN population of *A. valentinus* where different individuals belonged either to cluster II or III (Fig. 5A). Despite the near-perfect correspondence between *Anacyclus* species or clades and nuclear genetic clusters, three out of 47 populations deviated from this pattern: OUA from Morocco, was identified as *A. clavatus* but assigned to cluster I, while populations ELA and LAN from Algeria, identified as *A. valentinus* but assigned to cluster III. In the case of OUA, our results perfectly match the field notes, which mention a mixed population, likely introgressed by *A. homogamos*.

Based on the analysis of the intergenic spacers *rpL32-trnL* and *ycf1-rps15*, 17 haplotypes were identified across *Anacyclus* populations (Fig. 5B). Only one species, *A. pyrethrum*, was represented by a single haplotype, while the others ranged from two (*A. homogamos*, *A. linearilobus*, *A. monanthos*) to five haplotypes (*A. clavatus* and *A. valentinus*). The haplotypes observed in *A. homogamos* (K, L) and *A. monanthos* (E, F) were exclusive to these species. The other species displayed a combination of one or more exclusive haplotypes and shared haplotype(s). Our results show evidence of intra-population heterogeneity in five cases: CAR (A, H) and VAL (A, C) populations of *A. clavatus*, ALH (A, H) and NAD (A, H, P) populations of *A. valentinus*, and ALT (A, H), a mixed population of *A. clavatus* and *A. valentinus*.

### Plastome and 35S rDNA phylogenetic data

Complete plastomes for all *Anacyclus* species and *Heliocauta atlantica* were assembled, with their lengths ranging from 149,961 bp (*A. monanthos*) to 151,479 bp (*A. linearilobus*). The average read coverage per nucleotide ranged between 48.7 and 406.4, and the percentage of Ns in the complete plastome assemblies ranged between 0.02% and 0.31% (Table S4). The plastomes assembled in this study encode 80 CDS, 29 tRNAs and 4 rRNAs, giving a total of 113 genes (Table S5). Two canonical CDS genes were annotated as putative pseudogenes (Ψ) based on their altered structure: *ycf1* (IRa) and *rps19* (IRa). The gene order was conserved and no genome rearrangements were observed among the complete plastomes.

The phylogenetic trees based on plastome data obtained from Bayesian inference (BI) and maximum likelihood approaches generated almost identical topologies with generally high support values (Fig. 1B). The *Anacyclus* genus was recovered as monophyletic (PP = 1; BS = 100%), with *H. atlantica* as its sister species. *Anacyclus maroccanus* appears in an early-diverging position, being sister to the rest of the species in the genus (PP = 1; BS = 100%). Among the remaining *Anacyclus* species, *A. radiatus* and *A. homogamos* form a monophyletic clade (PP = 1; BS = 100%), sister to another clade clustering the rest of the species (PP = 1; BS = 100%). In this second clade, *A. monanthos* and *A. pyrethrum* appear in unresolved early-diverging branches, while *A. linearilobus*, *A. clavatus* and *A. valentinus* are grouped in a monophyletic lineage (PP = 0.99; BS = 67%). *Anacyclus linearilobus* is sister to the clade containing *A. clavatus* and *A. valentinus* (PP = 0.99; BS = 62%). The BI phylogenetic analysis of the dataset consisting of just the CDS regions (Fig. S5) produced a practically identical topology to that of the full dataset, only differing in the relative position of the unresolved branches bearing *A. monanthos* and *A. pyrethrum.*

The alignment of 35S rDNA sequences of *Anacyclus* species, *H. atlantica* and the outgroup consisted of 6,475 nucleotide positions, including 5′ETS, 18S gene, ITS1, 5.8S gene, ITS2 and 26S gene. The phylogenetic trees obtained from BI and maximum likelihood approaches generated similar topologies, but better support was observed in the BI tree (Fig. 1B). As in the plastome phylogenetic inference, the *Anacyclus* genus was recovered as monophyletic (PP = 1; BS = 100%), with *H. atlantica* as its sister species. *Anacyclus* species were split in two main clades. The first main clade (PP = 1; BS = 99%) contains *A. pyrethrum* as sister species to a subclade comprising *A. maroccanus* and *A. homogamos* (PP = 1; BS = 100%). The second main clade clusters *A. monanthos*, *A radiatus*, *A. valentinus*, *A. clavatus* and *A. linearilobus* (PP = 1; BS = 93%). Only the lineage grouping *A. valentinus*, *A. clavatus* and *A. linearilobus* shows statistical support (PP = 1) in this second main clade.

## Discussion

### Identity of Catalan specimens previously assigned to *A. homogamos*

The Catalan specimens identified as *A. homogamos* in Molero & Pyke (2019) were attributed to that species based on the absence of female-only florets (*i.e.,* hermaphroditic capitula). However, upon close examination of the herbarium sheets in the present study, inconspicuous ligulate female florets were observed, suggesting these specimens actually had a closer affinity to *A. valentinus* which is considered to be gynomonoecious (*i.e.,* individuals with capitula showing both female and hermaphrodite florets). This is consistent with the observation of Molero & Pyke (2019) who noted that individuals identified as *A. homogamos* were growing within dense populations of *A. valentinus*. Verifying the identity of specimens by dissecting capitula—and thus at least partially dismantling them until a ligulate floret is found (or not)—is not always feasible or desirable. Furthermore, a recent study of the genetic basis of sex expression in the genus led to the proposal of a model of genetic determination in which $ \frac{3}{4} $ of individuals in *A. valentinus* populations are expected to be gynomonoecious, while the remaining quarter—corresponding to recessive homozygotes at the second of two loci involved in gynomonoecy—present capitula with exclusively hermaphroditic florets (Álvarez et al., 2020). The presence of inconspicuous female florets can therefore no longer be considered a reliable character for identifying *A. valentinus*. It is worth noting that *A. valentinus* individuals with hermaphroditic capitula have rarely, if ever, been observed in experimental crosses or natural populations, possibly due to their low viability (Álvarez et al., 2020). These individuals may correspond to those observed by Molero & Pyke (2019). In the present study, we found four hermaphroditic *A. valentinus* capitula among the 23 dissected (Fig. 3D; Table S1). While the absence of female florets can no longer be used as a trait to distinguish *A. homogamos* from *A. valentinus*, our study shows that sequencing the ITS region or the plastid markers *rpL32–trnL* and *ycf1–rps15* is sufficient to differentiate the two species, as they belong to entirely distinct nuclear and plastid genetic groups (Fig. 5). It is also worth noting that the two species can be readily discriminated based on their genome sizes (Fig. 1A; Table 2).

### The valentinoid phenotype of the mixed Collserola (COL) population

In this study, we searched for individuals with a morphology similar to the Catalan specimens previously attributed to *A. homogamos*, and found them in the mixed *Anacyclus clavatus* and *A. valentinus* population COL. These individuals appear to represent an extreme within the broad phenotypic spectrum observed (Fig. 2B), and look like a reduced, stunted form of valentinoid phenotype (Table 1; Fig. 3E). However, in the ITS analysis, they do not group within the genetic cluster II of *A. valentinus*, but instead belong to nuclear genetic cluster III, together with *A. clavatus* and all individuals from the mixed *A. clavatus* and *A. valentinus* populations analysed in this study. This suggests they are most likely to be hybrids. It is generally expected that homoploid hybrids would exhibit morphologies intermediate between the parental species (*e.g.*, Pellicer et al., 2022) or resemble one parent more in cases of backcrossing with one of the parental species (*e.g.*, Pellicer et al., 2021). In this sense, this stunted valentinoid phenotype could represent a case of negative transgressive segregation and hybrid breakdown, whereby genetic incompatibilities between parental species are manifest phenotypically in the hybrid (Wong, Hiscock & Filatov, 2022; Wong et al., 2023). Prior studies have shown that species can persist in the face of asymmetric introgression from congeneric species, through the combined effects of selection on highly divergent regions (HDRs) and low recombination rates within contact zones (Gao et al., 2022; Xun et al., 2024). The phenotypic heterogeneity of the mixed populations (Fig. 2B; Figs. S1 & S3), coupled with the presence of hybrids with a stunted valentinoid phenotype and notably fewer florets than the parental species (*i.e.,* reduced fitness; Cerca et al., 2019; Table 1; Table S1), seems to suggest that HDRs between *A. clavatus* and *A. valentinus* do likely exist and may contain genes involved in capitulum traits. It is important to note that this phenotypic heterogeneity is not confined to mixed populations, as *A. valentinus* displays a notably broader morphospace than *A. clavatus* (Fig. 2B; Fig. S1). Furthermore, the two *A. valentinus* populations analysed in this study occupy non-overlapping morphospaces (Fig. S3B). These findings point to pronounced intra- and interpopulation morphological differentiation within *A. valentinus*, warranting further investigation through more comprehensive and targeted sampling.

### Genetic structure and genome size trends in *A. clavatus* and *A. valentinus*

The results of our ITS analysis are consistent with the genetic structure inferred from microsatellite data by Agudo et al. (2023) for *A. clavatus*, *A. homogamos*, *A. valentinus*, and mixed populations of *A. clavatus* and *A. valentinus*. Both studies indicate that *A. clavatus* and *A. valentinus* are more closely related to each other than to *A. homogamos*. Moreover, they concur in showing that individuals from mixed populations are genetically closer to *A. clavatus* than to *A. valentinus*, suggesting potential asymmetric introgression. However, unlike the microsatellite approach used by Agudo et al. (2023), our ITS-based analysis does not allow the detection of hybrids—except in the case mentioned in the previous section where individuals with a valentinoid phenotype group genetically with *A. clavatus*. In contrast, the microsatellite analysis revealed an intraspecific genetic structure within *A. clavatus*, identifying one cluster unique to the species, another shared with *A. valentinus*, and a third shared with mixed populations. Based on this finding, the existence of two distinct lineages within *A. clavatus* was proposed (Agudo et al., 2023). Our genome size analyses, combined with previous studies, also support the existence of two distinct *A. clavatus* groups, which follow a clear north–south (N/S) geographic pattern (Fig. 4A). Although it is not yet possible to determine whether these groups correspond to the genetic clusters identified through microsatellite analyses—since genetic and cytogenetic data from the same individuals are lacking—some hypotheses can already be ruled out. In particular, the explanation proposed by Agudo et al. (2019), suggesting that the smaller genome sizes observed in southern *A. clavatus* populations resulted from introgression with *A. valentinus* (which has a smaller genome), appears unsupported. Our expanded sampling, which includes a broader area of sympatry between *A. clavatus* and *A. valentinus*—beyond the Andalusian populations studied previously—shows indeed that the N/S pattern in genome size remains consistent along the Spanish Mediterranean coast. Populations from the northern half of the Spanish Mediterranean coast are particularly noteworthy, since, besides being an area where two species coexist, it includes *A. clavatus* populations with some of the highest recorded genome sizes and *A. valentinus* populations with some of the lowest genome sizes observed (Figs. 4A–4D). This is certainly not what one would anticipate in the context of homoploid hybridisation and introgression, where genome size differences between species are usually expected to be reduced rather than accentuated (*e.g.*, Pellicer et al., 2021). The contact zones between *A. clavatus* and *A. valentinus* may represent both a challenge and a potential solution for the long-term persistence of these species. While characterised by intense introgression, these zones also give rise to a remarkable diversity in 2C-values, occasionally decoupled from phenotype (Fig. 4A). This variation may act as a reservoir of genomic diversity upon which divergent selection can act. It is perhaps worth noting that this pattern recalls the one observed across the genus, where notable genome size differences are observed between *Anacyclus* species—particularly the most closely related ones (Fig. 1A). Such differences were suggested to result from homoploid hybridisation, where unequal chromosome rearrangements during the pairing of homeologous chromosomes from distinct genomes, lead to the loss or gain of chromosome fragments, and increased genome size divergence between species (Vitales et al., 2020). Further studies are needed to better understand the mechanisms by which homoploid hybridisation drives genome size divergence among closely related *Anacyclus* species, as well as the potential implications for their persistence in the presence of ongoing gene flow.

### Phylogenetic affinities across the genus

The genetic affinities among *Anacyclus* species recovered from the population-level ITS analysis (Fig. 5) and the species-level 35S rDNA phylogeny (Fig. 1) align closely with previous phylogenetic findings (Oberprieler, 2004; Vitales et al., 2018; Vitales et al., 2020). Our results show the same two main groups as previously reported, one comprising *A. clavatus*, *A. linearilobus*, *A. monanthos*, *A. radiatus* and *A. valentinus*, the other *A. homogamos*, *A. maroccanus* and *A. pyrethrum* (Oberprieler, 2004; Vitales et al., 2020). However, our study places *A. monanthos* as sister to all other species in the group, whereas Oberprieler (2004) recovered *A. clavatus* as its sister taxon, and Vitales et al. (2020) identified *A. radiatus* as its closest relative. It is worth noting that all three studies are based—entirely or primarily—on rDNA data, but differ substantially in both the composition of their molecular datasets (>6,000 bp of rDNA *vs.* ∼600 bp ITS) and taxonomic sampling strategies (one individual per species *vs.* populations).

Previous plastid phylogenetic reconstructions, based solely on the *psbA-trnH* marker (Vitales et al., 2018), resulted in a largely unresolved polytomy. Increasing the number of plastid genes analysed has substantially enhanced the phylogenetic resolution of the tree (Fig. 1B). This new reconstruction fundamentally reshapes all previously inferred relationships among taxa (Oberprieler, 2004; Vitales et al., 2018; Vitales et al., 2020). The only clade consistently recovered across our nuclear and plastid analyses is the one comprising *A. clavatus*, *A. linearilobus*, and *A. valentinus* (Fig. 1B). However, while 35S rDNA analyses identify *A. clavatus* and *A. linearilobus* as sister taxa, albeit with low supports, plastome analyses instead support a sister relationship between *A. clavatus* and *A. valentinus* (Fig. 1B). The clade comprising *A. homogamos*, *A. marcocanus*, and *A. pyrethrum* in the 35S rDNA phylogeny is not recovered in the plastome phylogeny. Instead, *A. homogamos* clusters with *A. radiatus*, *A. maroccanus* is placed as sister to all other *Anacyclus* species, and *A. pyrethrum* is recovered as sister to the clade comprising *A. clavatus*, *A. linearilobus*, *A. monanthos*, and *A. valentinus* (Fig. 1B).

Although some level of conflict between nuclear and plastid phylogenetic inferences was expected—given previous reports within the tribe (Criado-Ruiz et al., 2025) and intensive hybridisation among *Anacyclus* species—the extent of discordance observed here far exceeded our expectations. In light of this, it would be reasonable to conduct broader phylogenomic analyses based on more extensive population-level sampling to enable a more accurate interpretation of the results.

## Conclusions

Overall, our findings once again uncover the reticulate and dynamic nature of *Anacyclus* evolution. To advance understanding of such a complex, hybridogenic system, future research should integrate genetic, genomic, and phenotypic analyses from the same individuals, applied across the entire geographic range and at the population level. This integrative, individual-based framework is essential for unravelling evolutionary histories in plant lineages shaped by hybridisation and genome plasticity.

## Supplemental Information

10.7717/peerj.21638/supp-1 Supplemental Information 1 Morphological differentiation and trait associations among *Anacyclus clavatus* (blue), mixed (purple), and A. valentinus (red) populations The upper row shows group differences for each trait. The diagonal panels show trait density distributions, the lower triangle shows trait-by-trait scatterplots with group-specific trends, and the upper triangle reports Spearman’s rank correlation coefficients calculated for all individuals combined and separately by group. Significance codes apply to both pairwise comparisons and correlations: ns = non-significant; * = *p* ≤ 0.05; ** = *p* ≤ 0.01; *** = *p* ≤ 0.001.

10.7717/peerj.21638/supp-2 Supplemental Information 2 Image scans of specimens deposited in the BC and BCN herbaria and identified as *A. homogamos* A photograph showing the florets is provided, with a red arrow indicating the ligulate female florets.

10.7717/peerj.21638/supp-3 Supplemental Information 3 PCA of capitulum traits showing (A) the morphospaces occupied by *A. clavatus, A. valentinus* and mixed populations with individual identification and (B) the morphospaces occupied by each population with individual identification For further information about each numbered individual see Table S1.

10.7717/peerj.21638/supp-4 Supplemental Information 4 ITS sequence alignment Matrix of the ITS sequences

10.7717/peerj.21638/supp-5 Supplemental Information 5 Flow cytometry histogram of (A) specimen ALT 35-7, and (B) combined with the valentinoid individual ALT 35-4 which has the lowest genome size within the same population

10.7717/peerj.21638/supp-6 Supplemental Information 6 *rpL32-trnL* sequence alignment Matrix of the *rpL32-trnL* sequences

10.7717/peerj.21638/supp-7 Supplemental Information 7 Bayesian inference phylogenetic analysis of the plastome dataset consisting of CDS regions

10.7717/peerj.21638/supp-8 Supplemental Information 8 *ycf1-rps15* sequence alignment Matrix of the *ycf1-rps15* sequences

10.7717/peerj.21638/supp-9 Supplemental Information 9 Morphological characterisation of the capitula of individuals from *Anacyclus clavatus*, *A. valentinus* and mixed populations. SD, standard deviation. ⌀, diameter

10.7717/peerj.21638/supp-10 Supplemental Information 10 Genome size values for individuals. Pop., population; SD, standard deviation. Phenotypic data are provided for mixed populations where available

10.7717/peerj.21638/supp-11 Supplemental Information 11 Details of the samples used in the population genetic analyses

10.7717/peerj.21638/supp-12 Supplemental Information 12 Species information, SRA, plastome and 35S rDNA information for *Anacyclus* spp. and *Heliocauta atlantica*

10.7717/peerj.21638/supp-13 Supplemental Information 13 List of genes annotated in the studied plastid genomes of *Anacyclus* spp. and *Heliocauta atlantica*

10.7717/peerj.21638/supp-14 Supplemental Information 14 35S rDNA sequences deposited in GenBank (accession numbers PZ437183–PZ437192)

10.7717/peerj.21638/supp-15 Supplemental Information 15 Ycf1–rps15 sequences deposited in GenBank (accession numbers PX505180 –PX505254)

10.7717/peerj.21638/supp-16 Supplemental Information 16 ITS sequences deposited in GenBank (accession numbers PX447957 –PX448036)

10.7717/peerj.21638/supp-17 Supplemental Information 17 *rpL32F-trnL* sequences deposited in GenBank (accession numbers PX505105 –PX505179)

## Additional Information and Declarations

### Data Availability

The following information was supplied regarding data availability: The data is available in the Supplemental Files. The data is available at Zenodo: Hidalgo O & Pérez-Lorenzo I (2026). Genome size estimation of *Anacyclus* species and *Cota nigellifolia* (Asteraceae, Anthemideae) using flow cytometry. Zenodo. https://doi.org/10.5281/zenodo.18591815
The raw sequence data are available at GenBank: - ITS: PX447957 –PX448036
- *rpL32-trnL*: PX505105 –PX505179
- *ycf1–rps15*: PX505180 –PX505254.
