# Supplementary material for: Inside daisies’ evolutionary tangle: new insights into the hybridogenic genus Anacyclus (Asteraceae, Anthemideae)
Source: PeerJ. 2026 Aug 27;14:e21638. doi: 10.7717/peerj.21638 (PMC13528631; doi:10.7717/peerj.21638)
Supplement: Supplementary file 1 [file peerj-14-21638-s001.pdf]

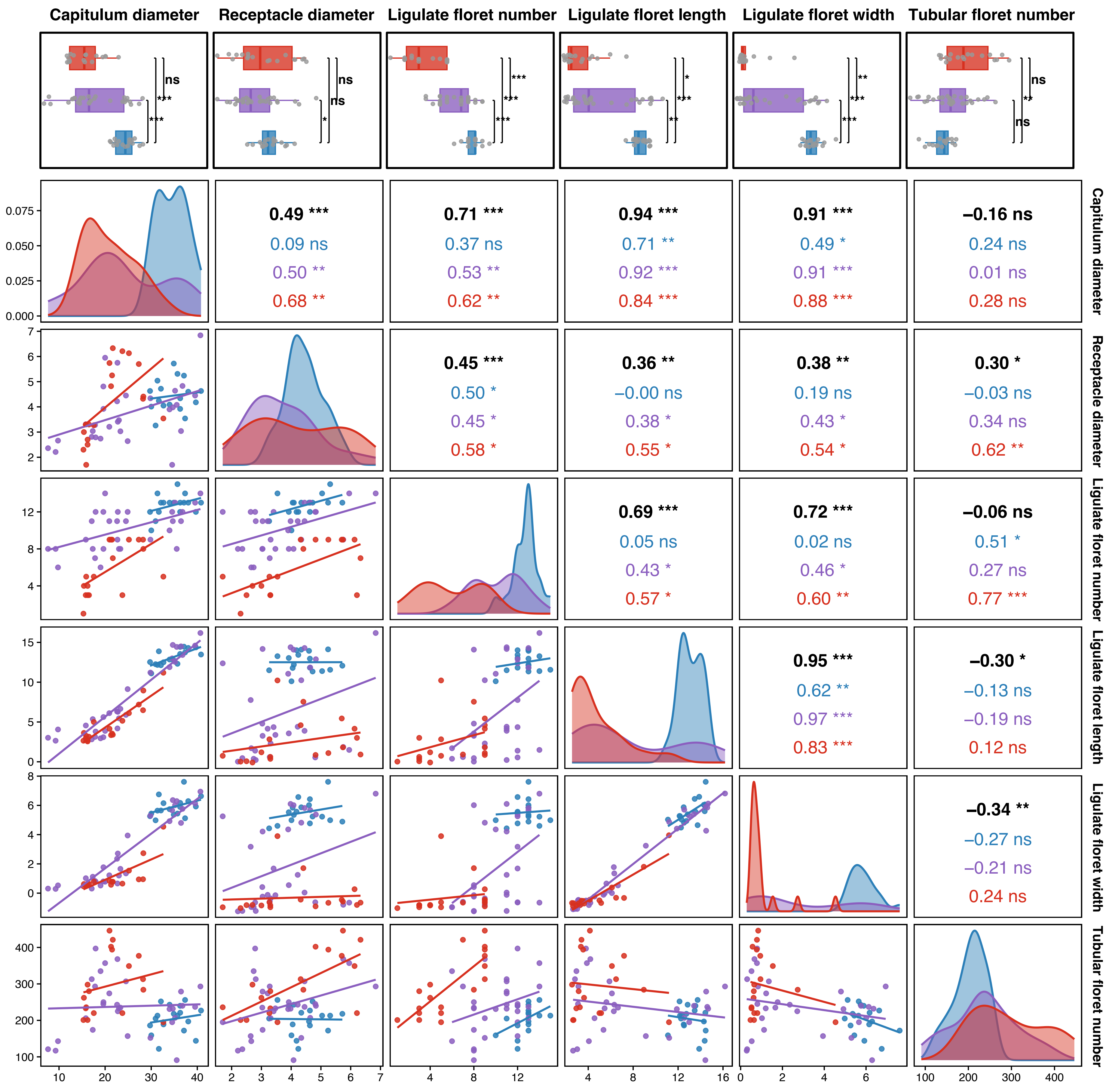

Capitulum diameter
Receptacle diameter
Ligulate floret number
Ligulate floret length
Ligulate floret width
Tubular floret number
0.49 ***
0.09 ns
0.50 **
0.68 **
0.71 ***
0.37 ns
0.53 **
0.62 **
0.94 ***
0.71 **
0.92 ***
0.84 ***
0.91 ***
0.49 *
0.91 ***
0.88 ***
−0.16 ns
0.24 ns
0.01 ns
0.28 ns
0.45 ***
0.50 *
0.45 *
0.58 *
0.36 **
−0.00 ns
0.38 *
0.55 *
0.38 **
0.19 ns
0.43 *
0.54 *
0.30 *
−0.03 ns
0.34 ns
0.62 **
0.69 ***
0.05 ns
0.43 *
0.57 *
0.72 ***
0.02 ns
0.46 *
0.60 **
−0.06 ns
0.51 *
0.27 ns
0.77 ***
0.95 ***
0.62 **
0.97 ***
0.83 ***
−0.30 *
−0.13 ns
−0.19 ns
0.12 ns
−0.34 **
−0.27 ns
−0.21 ns
0.24 ns
