# Supplementary material for: Inside daisies’ evolutionary tangle: new insights into the hybridogenic genus Anacyclus (Asteraceae, Anthemideae)
Source: PeerJ. 2026 Aug 27;14:e21638. doi: 10.7717/peerj.21638 (PMC13528631; doi:10.7717/peerj.21638)
Supplement: Supplementary file 2 [file peerj-14-21638-s002.pdf]

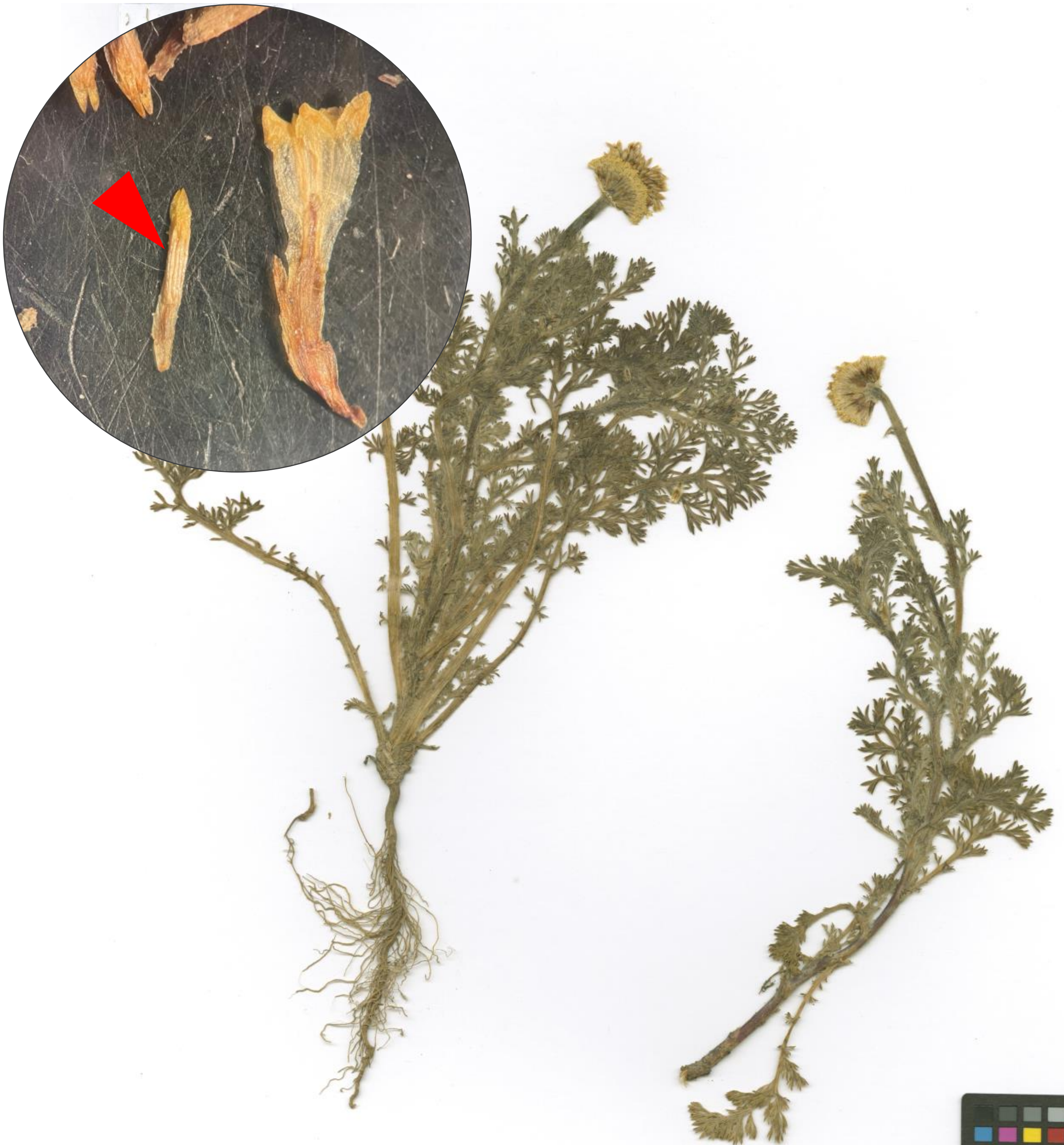

BCN 31856
Anacyclus valentinus L.
2 flores liguladas, guardadas en el sobre
Confirmat □
Revisor: I. Pérez-Lorenzo
Data: 13/05/22

0 1 2 3 4 5 6 7 8 9 10
cm
copyright reserved
Centre de Documentació de Biodiversitat Vegetal
CRAI-Universitat de Barcelona

BCN-31856
REAL JARDÍN BOTÁNICO DE MADRID. HERBARIO MA
Anacyclus homogamos (Maire) Humphries

Revisado I. Álvarez .... 15/9 de 20.10

BCN-31856
ex Herbarium BCF

Herbari Anna M. Rovira López

Estudi fitogeogràfic de les comarques catalanes compreses entre els Ports de Beseit, el riu Ebre i els límits aragonesos

BCN 31856 **BCF** (ex BCF 72008)
FACULTATIS PHARMACIÆ UNIVERSITATIS BARCINONENSIS
HERBARIUM

Anacyclus valentinus L.

Baix Ebre : Aldover (BF83). 10 - IV - 1987.

J. Molero & A. Rovira legerunt

BCN 31856
Cedoc Biodiversitat Vegetal. Barcelona

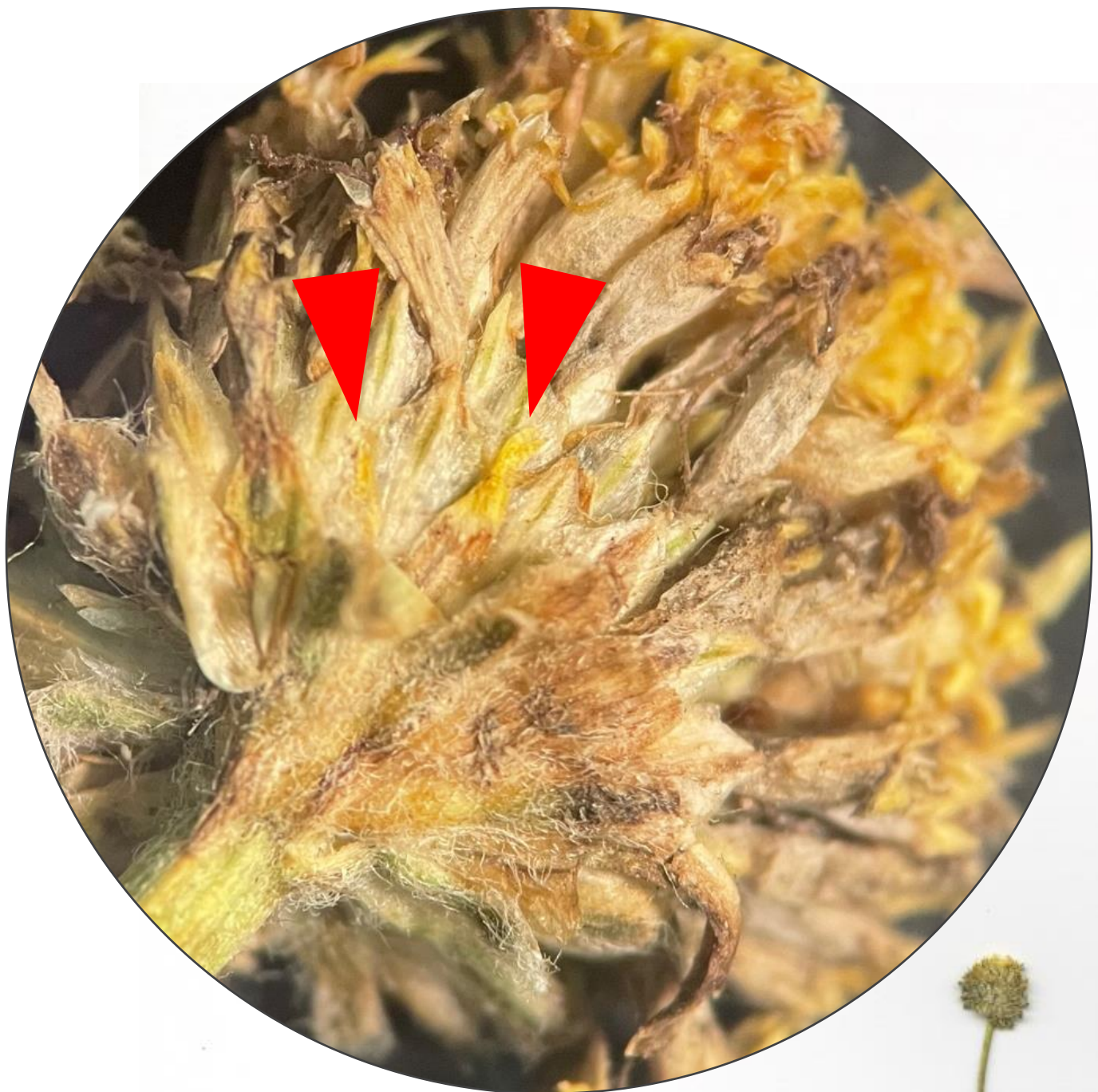

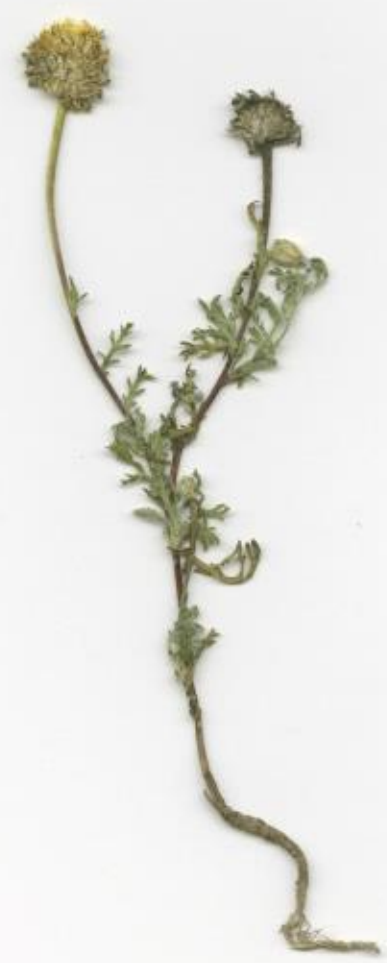

0 1 2 3 4 5 6 7 8 9 10
cm
copyright reserved

Centre de Documentació
de Biodiversitat Vegetal
CRAI-Universitat de Barcelona

Anacyclus homogamos (Maire)
Humphries
(= A. valentinus var. homogamos Maire)

Baix Camp: La Riba, vegetació
nitròfila al peu del riu Francolí,
CF47: N 41º 19,111' - E 1º 10,845' -
224 m. 5-09-2015, J. Molero

BCN. 125055

BCN 125055

Anacyclus valentinus L.
1. flor ligulada.
Confirmat ☐
Revisor: I. Pérez-Lorenzo
Data: 13/05/22

BCN 125055

CeDoc Biodiversitat Vegetal. Universitat de Barcelona

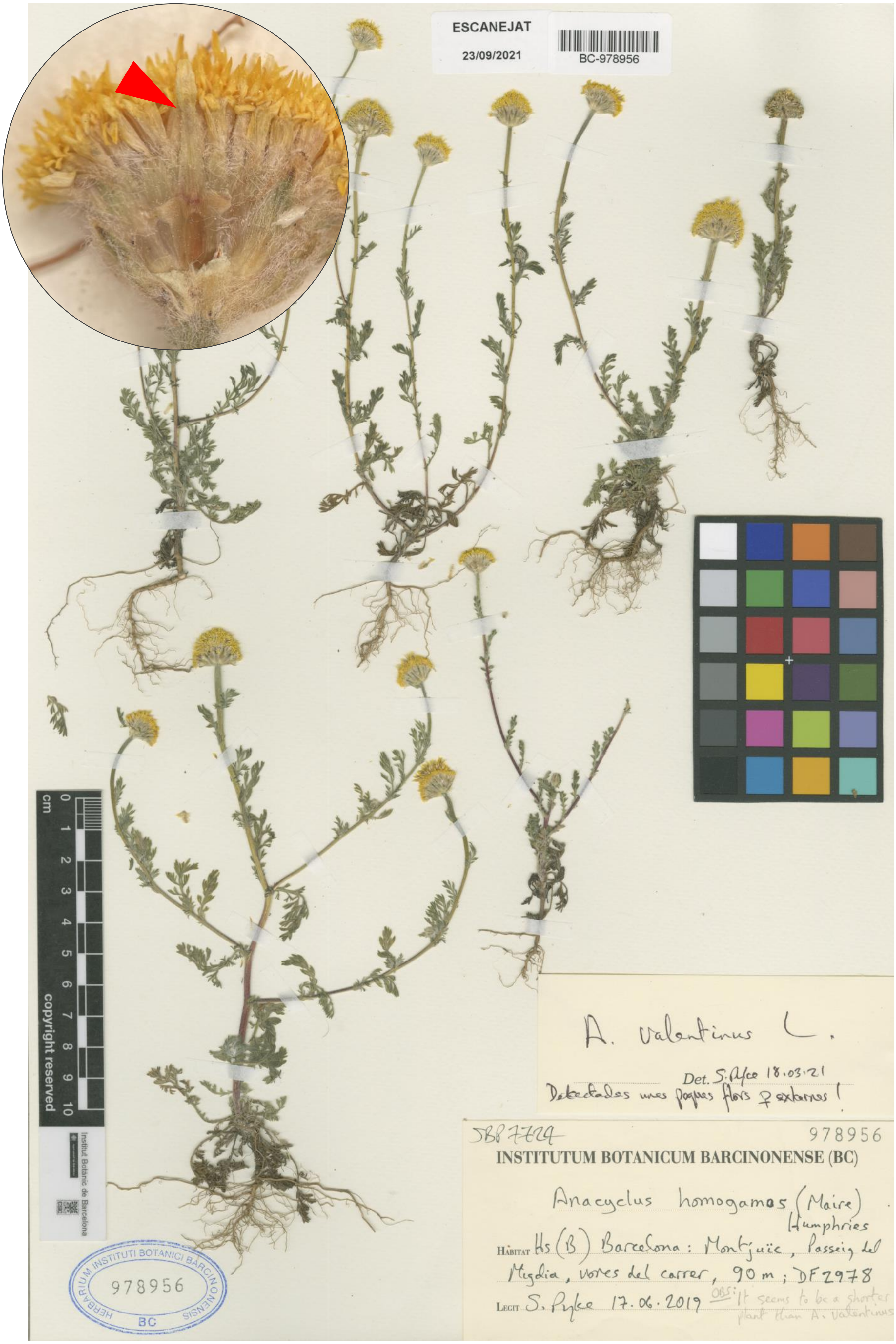
ESCANEJAT
23/09/2021
BC-978956
cm 0 1 2 3 4 5 6 7 8 9 10
copyright reserved
Institut Botànic de Barcelona
HERBARIUM INSTITUTI BOTANICI BARCINONENSIS
978956
BC
A. valentinus L.
Det. S. Pyke 18.03.21
Detectades unes poques flors ♀ externes!
SBP 7724
978956
INSTITUTUM BOTANICUM BARCINONENSE (BC)
Anacyclus homogamus (Maire) Humphries
HÀBITAT Hs (B) Barcelona: Montjuïc, Passeig del Migdia, vores del carrer, 90 m; DF2978
LEGIT S. Pyke 17.06.2019
OBS: It seems to be a shorter plant than A. valentinus
