# Supplementary material for: Inside daisies’ evolutionary tangle: new insights into the hybridogenic genus Anacyclus (Asteraceae, Anthemideae)
Source: PeerJ. 2026 Aug 27;14:e21638. doi: 10.7717/peerj.21638 (PMC13528631; doi:10.7717/peerj.21638)
Supplement: Supplementary file 3 [file peerj-14-21638-s003.pdf]

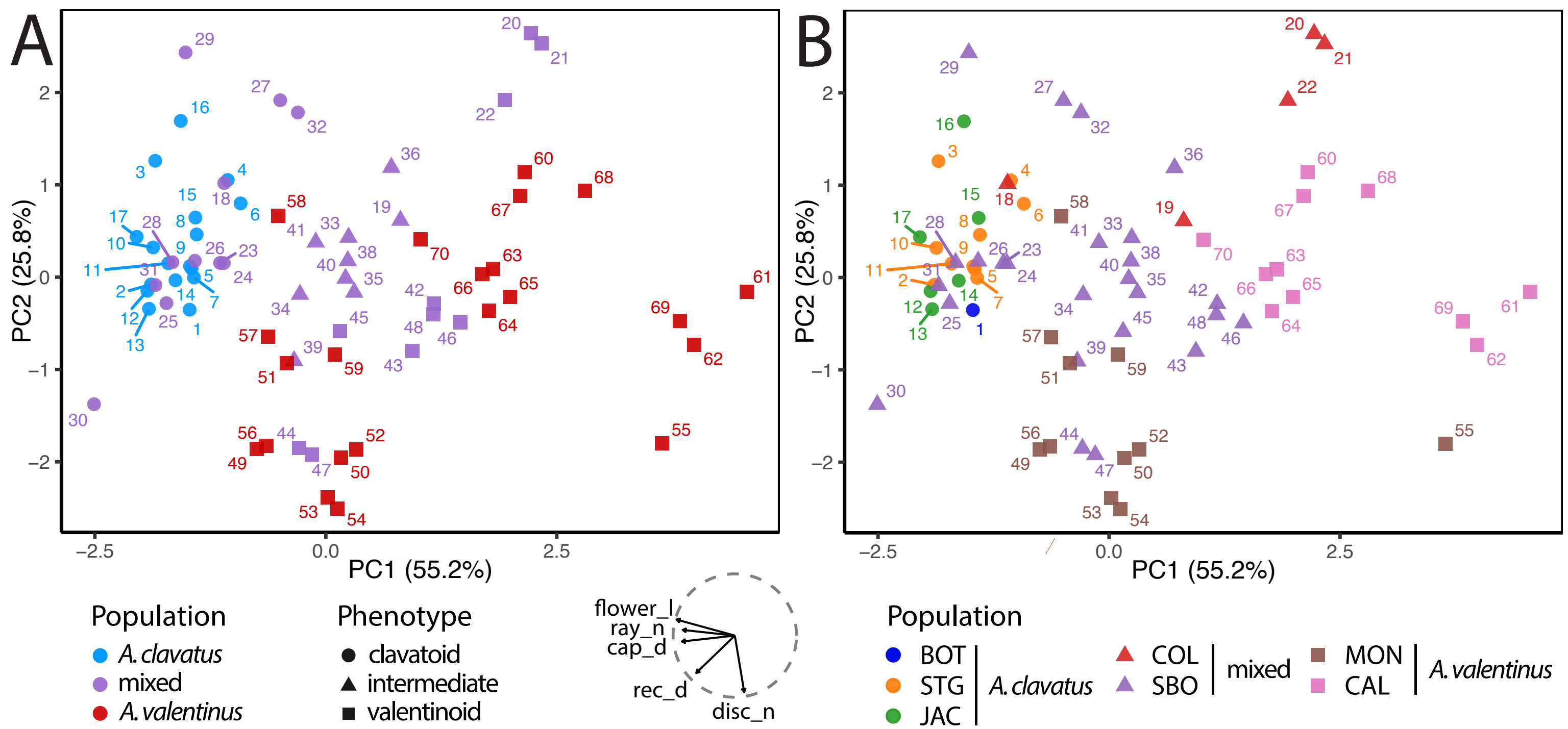

A
B
PC1 (55.2%)
PC2 (25.8%)
Population
A. clavatus
mixed
A. valentinus
Phenotype
clavatoid
intermediate
valentinoid
flower_l
ray_n
cap_d
rec_d
disc_n
BOT
STG
JAC
COL
SBO
MON
CAL
