# Supplementary material for: Inside daisies’ evolutionary tangle: new insights into the hybridogenic genus Anacyclus (Asteraceae, Anthemideae)
Source: PeerJ. 2026 Aug 27;14:e21638. doi: 10.7717/peerj.21638 (PMC13528631; doi:10.7717/peerj.21638)
Supplement: Supplementary file 4 [file peerj-14-21638-s005.pdf]

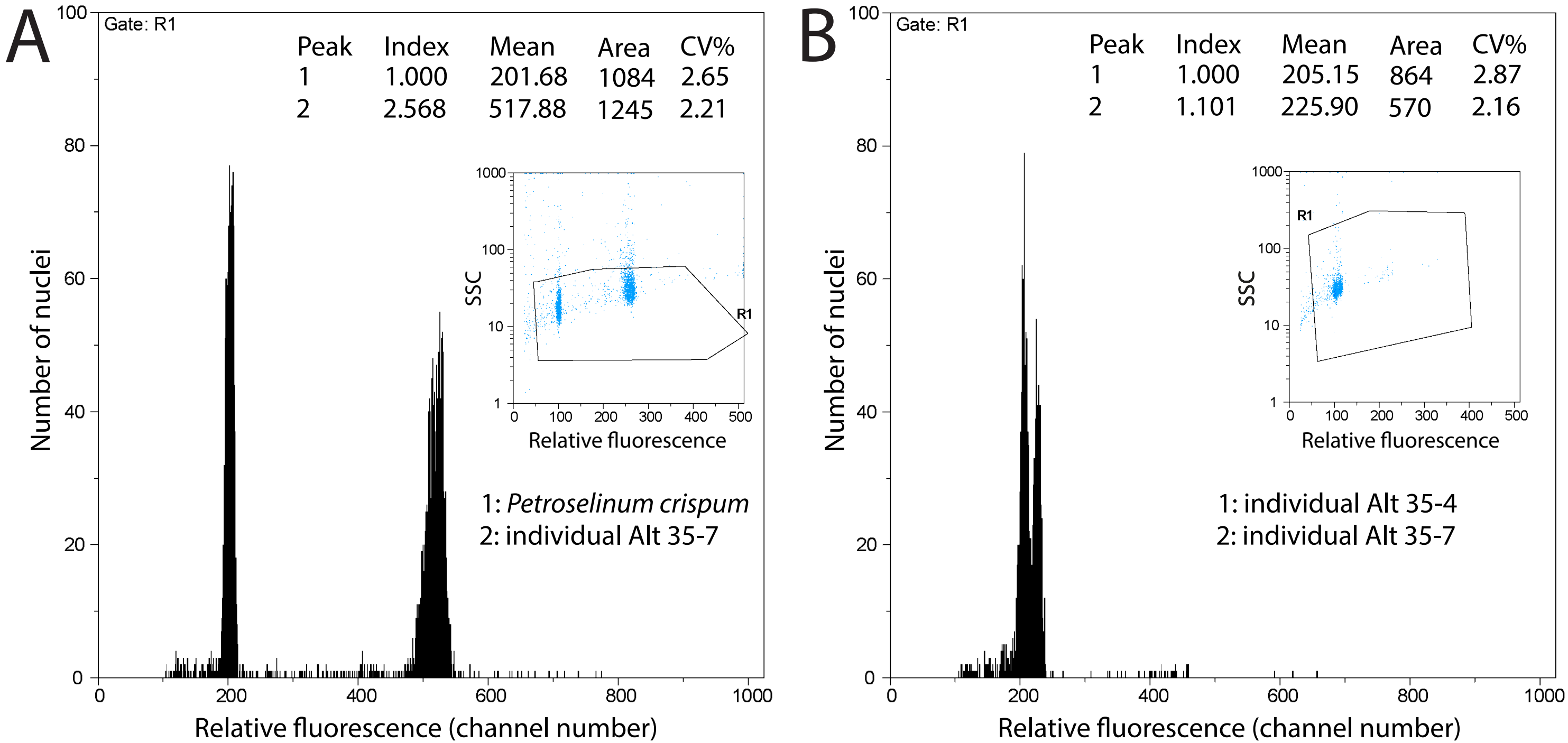
A
Gate: R1
Peak Index Mean Area CV%
1 1.000 201.68 1084 2.65
2 2.568 517.88 1245 2.21
SSC
R1
Relative fluorescence
1: Petroselinum crispum
2: individual Alt 35-7
Number of nuclei
Relative fluorescence (channel number)
B
Gate: R1
Peak Index Mean Area CV%
1 1.000 205.15 864 2.87
2 1.101 225.90 570 2.16
SSC
R1
Relative fluorescence
1: individual Alt 35-4
2: individual Alt 35-7
Number of nuclei
Relative fluorescence (channel number)
