# Supplementary material for: Inside daisies’ evolutionary tangle: new insights into the hybridogenic genus Anacyclus (Asteraceae, Anthemideae)
Source: PeerJ. 2026 Aug 27;14:e21638. doi: 10.7717/peerj.21638 (PMC13528631; doi:10.7717/peerj.21638)
Supplement: Supplementary file 5 [file peerj-14-21638-s007.pdf]

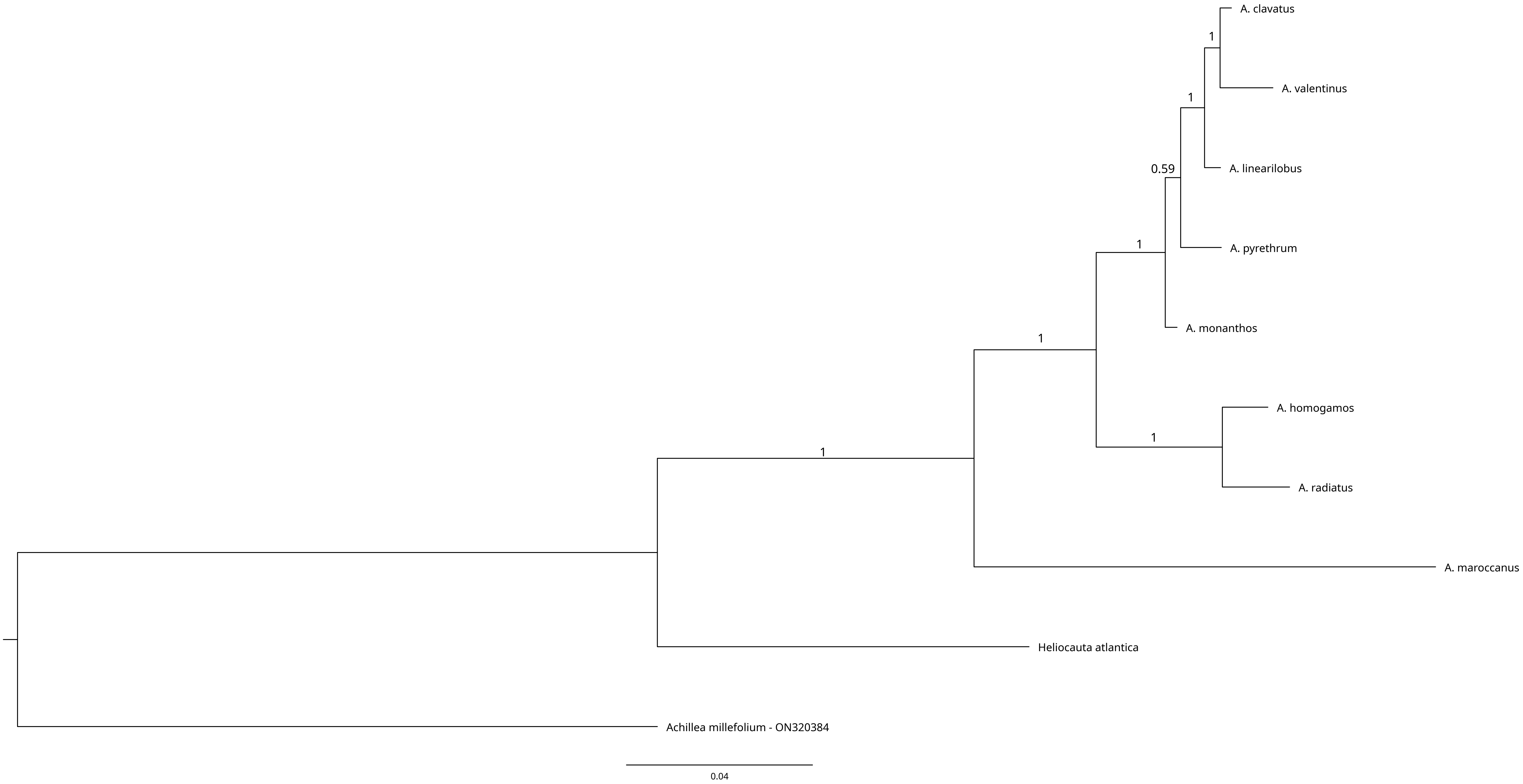
A. clavatus
1
A. valentinus
1
A. linearilobus
0.59
A. pyrethrum
1
A. monanthos
1
A. homogamos
1
1
A. radiatus
A. maroccanus
Heliocauta atlantica
Achillea millefolium - ON320384
0.04
