# Supplementary material for: Inside daisies’ evolutionary tangle: new insights into the hybridogenic genus Anacyclus (Asteraceae, Anthemideae)
Source: PeerJ. 2026 Aug 27;14:e21638. doi: 10.7717/peerj.21638 (PMC13528631; doi:10.7717/peerj.21638)
Supplement: Supplementary file 6 [file peerj-14-21638-s009.docx]

**Table S1.** Morphological characterisation of the capitula of individuals from *Anacyclus clavatus*, *A. valentinus* and mixed populations. SD: standard deviation.

| **Accession** | | | | | **Phenotype** | **Capitulum** | | **Ray florets** | | | | **Disc florets** |
| --- | --- | --- | --- | --- | --- | --- | --- | --- | --- | --- | --- | --- |
| **Taxon** | **Population** | **Pop. coord. GPS** | **Voucher** | **Individual (PCA)** | **(mixed pop. & trumpet)** | **⌀ (mm)** | **receptacle ⌀ (mm)** | **N** | **Flower length (SD) in mm** | **Limb width (SD) in mm** | **Colour** | **N** |
| *A. clavatus* | BOT | 41°00'57''N 0°23'08''E | OH 270 | 1 |  | 32.11 | 4.72 | 13 | 12.13 (0.27) | 5.65 (0.19) | white | 252 |
|  | STG | 43°40'51.0"N 4°26'00.3"E | OH 984 [20-V-24]-1 | 2 |  | 40.81 | 4.63 | 13 | 13.48 (0.26) | 6.63 (0.25) | white | 227 |
|  | STG | 43°40'51.0"N 4°26'00.3"E | OH 984 [20-V-24]-2 | 3 |  | 39.69 | 4.18 | 12 | 14.37 (0.27) | 5.92 (0.35) | white | 144 |
|  | STG | 43°40'51.0"N 4°26'00.3"E | OH 984 [20-V-24]-3 | 4 |  | 30.02 | 4.01 | 10 | 11.1 (0.2) | 5.44 (0.16) | white | 159 |
|  | STG | 43°40'51.0"N 4°26'00.3"E | OH 984 [20-V-24]-4 | 5 |  | 36.28 | 3.92 | 12 | 13.55 (0.44) | 5.89 (0.3) | white | 240 |
|  | STG | 43°40'51.0"N 4°26'00.3"E | OH 984 [20-V-24]-5 | 6 |  | 30.95 | 3.26 | 11 | 12.26 (0.6) | 4.98 (0.21) | white | 205 |
|  | STG | 43°40'51.0"N 4°26'00.3"E | OH 984 [20-V-24]-6 | 7 |  | 33.99 | 4.09 | 13 | 12.85 (0.36) | 5.74 (0.31) | white | 243 |
|  | STG | 43°40'51.0"N 4°26'00.3"E | OH 984 [20-V-24]-7 | 8 |  | 29.75 | 4.63 | 12 | 12.53 (0.3) | 5.26 (0.14) | white | 186 |
|  | STG | 43°40'51.0"N 4°26'00.3"E | OH 984 [20-V-24]-8 | 9 |  | 37.35 | 3.54 | 14 | 13.74 (0.4) | 5.12 (0.2) | white | 256 |
|  | STG | 43°40'51.0"N 4°26'00.3"E | OH 984 [20-V-24]-9 | 10 |  | 38.04 | 4.6 | 13 | 14.3 (0.31) | 6.29 (0.23) | white | 197 |
|  | STG | 43°40'51.0"N 4°26'00.3"E | OH 984 [20-V-24]-10 | 11 |  | 36.29 | 4.34 | 13 | 14.43 (0.4) | 6.17 (0.21) | white | 221 |
|  | JAC | 42°34'14.1"N 0°34'13.2"W | ILP [18-VI-24]1-1 | 12 |  | 35.7 | 5.31 | 15 | 12.31 (1.1) | 5.44 (0.57) | white | 213 |
|  | JAC | 42°34'14.1"N 0°34'13.2"W | ILP [18-VI-24]1-2 | 13 |  | 34.9 | 5.72 | 13 | 12.74 (0.35) | 5.36 (0.31) | white | 218 |
|  | JAC | 42°34'14.1"N 0°34'13.2"W | ILP [18-VI-24]1-3 | 14 |  | 31.36 | 5.04 | 14 | 12.17 (0.62) | 5.64 (0.35) | white | 213 |
|  | JAC | 42°34'14.1"N 0°34'13.2"W | ILP [18-VI-24]1-4 | 15 |  | 32.67 | 4.05 | 13 | 12.5 (0.48) | 6.15 (0.58) | white | 189 |
|  | JAC | 42°34'14.1"N 0°34'13.2"W | ILP [18-VI-24]1-5 | 16 |  | 31.66 | 4.24 | 12 | 13.88 (0.41) | 6.75 (0.49) | white | 122 |
|  | JAC | 42°34'14.1"N 0°34'13.2"W | ILP [29-V-24]1-1 | 17 |  | 37.11 | 5.24 | 13 | 14.46 (0.52) | 7.61 (0.25) | white | 172 |
| mixed | COL | 41°24'23''N 2°06'06''E | OH 692 | 18 | clavatoid | 29.83 | 4.34 | 9 | 11.21 (0.35) | 5.3 (0.23) | white | 152 |
|  | COL | 41°24'23''N 2°06'06''E | OH 686 | 19 | intermediate | 15.82 | 3.31 | 9 | 4.56 (0.37) | 1.02 (0.06) | white | 206 |
|  | COL | 41°24'23''N 2°06'06''E | IPL 2 | 20 | valentinoid | 9.26 | 2.21 | 8 | 2.65 (0.06) | 0.29 (0.05) | yellow | 117 |
|  | COL | 41°24'23''N 2°06'06''E | OH 690 | 21 | valentinoid | 7.62 | 2.36 | 8 | 3.02 (0.09) | 0.31 (0.03) | yellow | 121 |
|  | COL | 41°24'23''N 2°06'06''E | OH 688 | 22 | valentinoid | 9.78 | 2.66 | 6 | 4.06 (0.12) | 0.53 (0.05) | yellow | 143 |
|  | SBO | 41°20'31.0"N 2°02'02.2"E | ILP [10-V-25]1-1 | 23 | clavatoid | 33.98 | 3.9 | 10 | 11.89 (0.29) | 4.92 (0.32) | white | 231 |
|  | SBO | 41°20'31.0"N 2°02'02.2"E | ILP [10-V-25]1-2 | 24 | clavatoid | 36.47 | 3.04 | 11 | 14.5 (0.6) | 4.8 (0.26) | white | 279 |
|  | SBO | 41°20'31.0"N 2°02'02.2"E | ILP [10-V-25]1-3 | 25 | clavatoid | 36.79 | 4.83 | 12 | 13.42 (0.51) | 5.76 (0.37) | white | 240 |
|  | SBO | 41°20'31.0"N 2°02'02.2"E | ILP [10-V-25]1-4 | 26 | clavatoid | 34.69 | 3.98 | 11 | 14.68 (0.51) | 6.36 (0.4) | white | 232 |
|  | SBO | 41°20'31.0"N 2°02'02.2"E | ILP [10-V-25]1-5 | 27 | clavatoid | 34.55 | 1.7 | 12 | 12.99 (0.43) | 5.66 (0.3) | white | 195 |
|  | SBO | 41°20'31.0"N 2°02'02.2"E | ILP [10-V-25]1-6 | 28 | clavatoid | 35.18 | 4.64 | 13 | 12.55 (0.51) | 6.01 (0.25) | white | 207 |
|  | SBO | 41°20'31.0"N 2°02'02.2"E | ILP [10-V-25]1-7 | 29 | clavatoid | 35.54 | 4.08 | 8 | 14.43 (0.58) | 6.29 (0.32) | white | 91 |
|  | SBO | 41°20'31.0"N 2°02'02.2"E | ILP [10-V-25]1-8 | 30 | clavatoid | 40.68 | 6.84 | 14 | 16.18 (0.29) | 6.94 (0.47) | white | 293 |
|  | SBO | 41°20'31.0"N 2°02'02.2"E | ILP [10-V-25]1-9 | 31 | clavatoid | 39.53 | 4.45 | 13 | 14.6 (0.48) | 6.13 (0.16) | white | 237 |
|  | SBO | 41°20'31.0"N 2°02'02.2"E | ILP [10-V-25]1-10 | 32 | clavatoid | 24.89 | 2.64 | 12 | 9.13 (0.21) | 3.94 (0.25) | white | 157 |
|  | SBO | 41°20'31.0"N 2°02'02.2"E | ILP [10-V-25]2-1 | 33 | intermediate | 23.32 | 3.03 | 8 | 6.65 (0.29) | 1.66 (0.2) | white | 235 |
|  | SBO | 41°20'31.0"N 2°02'02.2"E | ILP [10-V-25]2-2 | 34 | intermediate | 22.6 | 4.22 | 11 | 6.32 (0.17) | 2.35 (0.41) | white | 243 |
|  | SBO | 41°20'31.0"N 2°02'02.2"E | ILP [10-V-25]2-3 | 35 | intermediate | 19.64 | 3.92 | 8 | 5.62 (0.17) | 1.77 (0.07) | yellow | 250 |
|  | SBO | 41°20'31.0"N 2°02'02.2"E | ILP [10-V-25]2-4 | 36 | intermediate | 17.32 | 3.04 | 8 | 5.36 (0.24) | 1.12 (0.13) | white | 174 |
|  | SBO | 41°20'31.0"N 2°02'02.2"E | ILP [10-V-25]2-5 | 37 | intermediate | 17.32 | 2.92 | 11 | 4.11 (0.37) | 1.56 (0.35) | white | NA |
|  | SBO | 41°20'31.0"N 2°02'02.2"E | ILP [10-V-25]2-6 | 38 | intermediate | 23 | 3.47 | 8 | 5.46 (0.41) | 1.2 (0.08) | white | 233 |
|  | SBO | 41°20'31.0"N 2°02'02.2"E | ILP [10-V-25]2-7 | 39 | intermediate | 24.52 | 4.45 | 11 | 5.89 (0.21) | 1.35 (0.03) | white | 307 |
|  | SBO | 41°20'31.0"N 2°02'02.2"E | ILP [10-V-25]2-8 | 40 | intermediate | 20.91 | 3.21 | 11 | 6.11 (0.32) | 1.22 (0.24) | white | 274 |
|  | SBO | 41°20'31.0"N 2°02'02.2"E | ILP [10-V-25]2-9 |  | intermediate | 19.79 | 3.91 | 12 | NA | NA | NA | 261 |
|  | SBO | 41°20'31.0"N 2°02'02.2"E | ILP [10-V-25]2-10 | 41 | intermediate | 22.76 | 3.41 | 12 | 6.28 (0.44) | 2.71 (0.3) | white | 225 |
|  | SBO | 41°20'31.0"N 2°02'02.2"E | ILP [10-V-25]3-1 | 42 | valentinoid | 17.1 | 2.81 | 11 | 2.82 (0.21) | 0.31 (0.05) | NA | 313 |
|  | SBO | 41°20'31.0"N 2°02'02.2"E | ILP [10-V-25]3-2 |  | valentinoid | 16.59 | 3.42 | 12 | NA | NA | NA | 210 |
|  | SBO | 41°20'31.0"N 2°02'02.2"E | ILP [10-V-25]3-3 | 43 | valentinoid | 17.94 | 2.79 | 12 | 3.8 (NA) | 0.79 (NA) | yellow | 397 |
|  | SBO | 41°20'31.0"N 2°02'02.2"E | ILP [10-V-25]3-4 |  | valentinoid | 18.14 | 4.36 | 10 | NA | NA | NA | 311 |
|  | SBO | 41°20'31.0"N 2°02'02.2"E | ILP [10-V-25]3-5 | 44 | valentinoid | 20 | 5.95 | 14 | 3.88 (NA) | 0.78 (NA) | NA | 357 |
|  | SBO | 41°20'31.0"N 2°02'02.2"E | ILP [10-V-25]3-6 | 45 | valentinoid | 19.62 | 4,81 | 12 | 3.09 (0.12) | 0.8 (0.16) | yellow | 245 |
|  | SBO | 41°20'31.0"N 2°02'02.2"E | ILP [10-V-25]3-7 | 46 | valentinoid | 19.01 | 2.74 | 6 | 2.95 (0.03) | 0.52 (0.1) | yellow | 335 |
|  | SBO | 41°20'31.0"N 2°02'02.2"E | ILP [10-V-25]3-8 |  | valentinoid | 19.78 | 4.75 | 8 | NA | NA | NA | 337 |
|  | SBO | 41°20'31.0"N 2°02'02.2"E | ILP [10-V-25]3-9 | 47 | valentinoid | 22.68 | 5.75 | 9 | 4.19 (0.16) | 0.66 (0.19) | yellow | 368 |
|  | SBO | 41°20'31.0"N 2°02'02.2"E | ILP [10-V-25]3-10 | 48 | valentinoid | 17.82 | 3.28 | 7 | 3.13 (0.13) | 0.83 (0.19) | yellow | 293 |
| *A. valentinus* | MON | 41°21'44''N 2°09'34''E | OH 1057-1 | 49 |  | 27.31 | 5.7 | 9 | 7.19 (0.04) | 0.94 (0.26) | yellow | 377 |
|  | MON | 41°21'44''N 2°09'34''E | OH 1057-2 | 50 |  | 21.51 | 5.25 | 9 | 3.55 (0.29) | 0.77 (0.17) | yellow | 394 |
|  | MON | 41°21'44''N 2°09'34''E | OH 1057-3 | 51 | trumpet | 28.23 | 4.32 | 9 | 6.48 (0.26) | 0.95 (0.17) | yellow | 313 |
|  | MON | 41°21'44''N 2°09'34''E | OH 1057-4 | 52 |  | 21.29 | 4.82 | 9 | 3.35 (0.17) | 0.57 (0.09) | yellow | 402 |
|  | MON | 41°21'44''N 2°09'34''E | OH 1057-5 | 53 |  | 20.95 | 5.74 | 9 | 4.15 (0.17) | 0.84 (0.11) | yellow | 446 |
|  | MON | 41°21'44''N 2°09'34''E | OH 1057-6 | 54 |  | 21.67 | 6.33 | 7 | 3.4 (0.23) | 0.78 (0.14) | yellow | 421 |
|  | MON | 41°21'44''N 2°09'34''E | OH 1057-7 |  |  | 20.37 | 4.61 | 7 | NA | NA | yellow | 412 |
|  | MON | 41°21'44''N 2°09'34''E | OH 1057-8 | 55 |  | 18.51 | 3.9 | 0 | _ | _ | _ | 343 |
|  | MON | 41°21'44''N 2°09'34''E | OH 1057-9 | 56 |  | 25.17 | 6.13 | 9 | 6.11 (0.51) | 1.54 (0.25) | yellow | 349 |
|  | MON | 41°21'44''N 2°09'34''E | OH 1057-10 | 57 |  | 28.3 | 4.41 | 8 | 8.95 (0.66) | 2.73 (0.24) | yellow | 284 |
|  | MON | 41°21'44''N 2°09'34''E | OH 1057-11 | 58 |  | 32.64 | 3.54 | 5 | 11.19 (0.32) | 4.54 (0.47) | yellow | 195 |
|  | MON | 41°21'44''N 2°09'34''E | JBB | 59 |  | 23.75 | 6.21 | 3 | 5.15 (0.22) | 0.65 (0.08) | yellow | 220 |
|  | CAL | 41°38'26.2"N 2°10'23.1"E | IPL [8-VI-25]1 | 60 |  | 15.9 | 1.7 | 5 | 3.26 (NA) | 0.52 (NA) | yellow | 236 |
|  | CAL | 41°38'26.2"N 2°10'23.1"E | IPL [8-VI-25]2 | 61 |  | 17.1 | 1.8 | 0 | _ | _ | _ | 291 |
|  | CAL | 41°38'26.2"N 2°10'23.1"E | IPL [8-VI-25]3 | 62 |  | 17.7 | 2.9 | 0 | _ | _ | _ | 274 |
|  | CAL | 41°38'26.2"N 2°10'23.1"E | IPL [8-VI-25]4 | 63 |  | 15.8 | 3.3 | 3 | 2.94 (0.25) | 0.52 (0.04) | yellow | 233 |
|  | CAL | 41°38'26.2"N 2°10'23.1"E | IPL [8-VI-25]5 | 64 |  | 16.5 | 3.3 | 3 | 3.15 (0.19) | 0.63 (0.01) | yellow | 280 |
|  | CAL | 41°38'26.2"N 2°10'23.1"E | IPL [8-VI-25]6 | 65 |  | 16.2 | 2.7 | 4 | 2.56 (0.17) | 0.65 (0.12) | yellow | 298 |
|  | CAL | 41°38'26.2"N 2°10'23.1"E | IPL [8-VI-25]7 | 66 |  | 15.4 | 3 | 4 | 3.63 (0.32) | 0.62 (0.01) | yellow | 262 |
|  | CAL | 41°38'26.2"N 2°10'23.1"E | IPL [8-VI-25]8 | 67 |  | 16.2 | 2.5 | 3 | 2.69 (0.2) | 0.64 (0.01) | yellow | 201 |
|  | CAL | 41°38'26.2"N 2°10'23.1"E | IPL [8-VI-25]9 | 68 |  | 15.3 | 2.3 | 1 | 2.65 (NA) | 0.47 (NA) | yellow | 201 |
|  | CAL | 41°38'26.2"N 2°10'23.1"E | IPL [8-VI-25]10 | 69 |  | 16.6 | 3.4 | 0 | _ | _ | _ | 224 |
|  | CAL | 41°38'27''N 2°10'05''E | GR 560 | 70 |  | 17.55 | 3,27 | 5 | 5 (0.14) | 0.8 (0.15) | yellow | 220 |
