# Supplementary material for: Inside daisies’ evolutionary tangle: new insights into the hybridogenic genus Anacyclus (Asteraceae, Anthemideae)
Source: PeerJ. 2026 Aug 27;14:e21638. doi: 10.7717/peerj.21638 (PMC13528631; doi:10.7717/peerj.21638)
Supplement: Supplementary file 7 [file peerj-14-21638-s010.docx]

**Table S2.** Genome size values for individuals. Pop.: population; SD: standard deviation. Phenotypic data are provided for mixed populations where available.

| Species in population | Pop. coord. GPS/Loc. | Code pop. | Population | Individual | 2C(pg) | SD | CVstd (%) | Cvplt (%) | Standard |
| --- | --- | --- | --- | --- | --- | --- | --- | --- | --- |
| *Anacyclus clavatus* | 41°00'57''N 0°23'08''E | BOT | OH 270 | OH 270/1 | 10.73 | 0.02 | 2.68 | 2.84 | Petroselinum |
| *Anacyclus clavatus* | 41°00'57''N 0°23'08''E | BOT | OH 270 | OH 270/2 | 10.74 | 0.02 | 2.82 | 2.96 | Petroselinum |
| *Anacyclus clavatus* | 41°00'57''N 0°23'08''E | BOT | OH 270 | OH 270/3 | 10.80 | 0.02 | 2.77 | 2.76 | Petroselinum |
| *Anacyclus clavatus* | 42°7'27.2''N 2°57'23.5''E | CAM | IA 2050 | IA2050 | 10.67 | 0.02 | 2.11 | 2.13 | Petroselinum |
| *Anacyclus clavatus* | 36°41'49''N 3°27'33''W | CAR | AA 1 | trum B2 | 9.52 | 0.01 | 2.41 | 2.12 | Petroselinum |
| *Anacyclus clavatus* | 36°41'49''N 3°27'33''W | CAR | AA 1 | trum B2 (1) | 9.72 | 0.03 | 2.14 | 2.01 | Petroselinum |
| *Anacyclus clavatus* | 36°41'49''N 3°27'33''W | CAR | AA 1 | trum B2 (2) | 9.57 | 0.01 | 2.38 | 2.06 | Petroselinum |
| *Anacyclus clavatus* | 36°41'49''N 3°27'33''W | CAR | AA 1 | trum B2 (3) | 9.53 | 0.00 | 1.81 | 1.52 | Petroselinum |
| *Anacyclus clavatus* | 36°41'49''N 3°27'33''W | CAR | AA 1 | trum B2 (4) | 9.63 | 0.00 | 2.31 | 1.89 | Petroselinum |
| *Anacyclus clavatus* | 36°41'49''N 3°27'33''W | CAR | AA 1 | trum B2 (5) | 9.62 | 0.01 | 2.45 | 2.06 | Petroselinum |
| *Anacyclus clavatus* | 36°41'49''N 3°27'33''W | CAR | AA 1 | trum B2 (6) | 9.69 | 0.00 | 2.05 | 1.75 | Petroselinum |
| *Anacyclus clavatus* | 42°16'3.75''N 4°24'19.23''W | FRO | IA 2073 | IA2073-1 | 10.9 | 0.02 | 1.83 | 2.06 | Petroselinum |
| *Anacyclus clavatus* | 42°16'3.75''N 4°24'19.23''W | FRO | IA 2073 | IA2073-10 | 10.56 | 0.03 | 2.14 | 2.25 | Petroselinum |
| *Anacyclus clavatus* | 42°16'3.75''N 4°24'19.23''W | FRO | IA 2073 | IA2073-12 | 10.81 | 0.01 | 2.25 | 2.13 | Petroselinum |
| *Anacyclus clavatus* | 42°16'3.75''N 4°24'19.23''W | FRO | IA 2073 | IA2073-2 | 10.62 | 0.01 | 1.89 | 1.9 | Petroselinum |
| *Anacyclus clavatus* | 42°16'3.75''N 4°24'19.23''W | FRO | IA 2073 | IA2073-5 | 10.51 | 0 | 2.35 | 2.54 | Petroselinum |
| *Anacyclus clavatus* | 42°16'3.75''N 4°24'19.23''W | FRO | IA 2073 | IA2073-6 | 10.62 | 0.02 | 2.27 | 2.74 | Petroselinum |
| *Anacyclus clavatus* | 42°16'3.75''N 4°24'19.23''W | FRO | IA 2073 | IA2073-7 | 10.63 | 0.02 | 2.25 | 2.35 | Petroselinum |
| *Anacyclus clavatus* | 40°19'59.9''N 9°31'53.5''E | ITA | LM 5110 | LM5110-14 | 10.84 | 0.02 | 1.84 | 1.91 | Petroselinum |
| *Anacyclus clavatus* | 40°19'59.9''N 9°31'53.5''E | ITA | LM 5110 | LM5110-15 | 10.9 | 0.02 | 1.87 | 2.12 | Petroselinum |
| *Anacyclus clavatus* | 40°19'59.9''N 9°31'53.5''E | ITA | LM 5110 | LM5110-16 | 10.75 | 0.01 | 1.68 | 2.04 | Petroselinum |
| *Anacyclus clavatus* | 36°43'30.1''N 3°34'50.4''W | SAL | IA 2135 | IA2135-3 | 9.65 | 0 | 2.4 | 2.59 | Petroselinum |
| *Anacyclus clavatus* | 36°43'30.1''N 3°34'50.4''W | SAL | IA 2135 | IA2135-4 | 9.56 | 0.01 | 2.72 | 2.83 | Petroselinum |
| *Anacyclus clavatus* | 36°43'30.1''N 3°34'50.4''W | SAL | IA 2135 | IA2135-5 | 9.51 | 0 | 2.05 | 2.87 | Petroselinum |
| *Anacyclus clavatus* | 36°43'30.1''N 3°34'50.4''W | SAL | IA 2135 | IA2135-6 | 9.43 | 0.02 | 2.65 | 2.95 | Petroselinum |
| *Anacyclus clavatus* | 36°43'30.1''N 3°34'50.4''W | SAL | IA 2135 | IA2135-7 | 9.56 | 0 | 2.06 | 2.59 | Petroselinum |
| *Anacyclus clavatus* | 43°40'51.0"N 4°26'00.3"E | STG | OH 984 | OH 984[23-V-24]-1 | 10.62 | 0.06 | 2.87 | 2.81 | Petroselinum |
| *Anacyclus clavatus* | 43°40'51.0"N 4°26'00.3"E | STG | OH 984 | OH 984[23-V-24]-2 | 10.48 | 0.02 | 2.78 | 2.71 | Petroselinum |
| *Anacyclus clavatus* | 43°40'51.0"N 4°26'00.3"E | STG | OH 984 | OH 984[23-V-24]-3 | 10.60 | 0.02 | 2.61 | 2.44 | Petroselinum |
| *Anacyclus clavatus* | 43°40'51.0"N 4°26'00.3"E | STG | OH 984 | OH 984[23-V-24]-4 | 10.49 | 0.03 | 2.63 | 2.72 | Petroselinum |
| *Anacyclus clavatus* | 43°40'51.0"N 4°26'00.3"E | STG | OH 984 | OH 984[23-V-24]-5 | 10.69 | 0.02 | 2.41 | 2.40 | Petroselinum |
| *Anacyclus clavatus* | 42°12'8''N 1°38'43''W | VAL | IA 2069 | Val (1) | 10.61 | 0.01 | 1.99 | 1.85 | Petroselinum |
| *Anacyclus clavatus* | 42°12'8''N 1°38'43''W | VAL | IA 2069 | Val (10) | 10.61 | 0.02 | 2.19 | 1.94 | Petroselinum |
| *Anacyclus clavatus* | 42°12'8''N 1°38'43''W | VAL | IA 2069 | Val (2) | 10.60 | 0.01 | 1.91 | 1.89 | Petroselinum |
| *Anacyclus clavatus* | 42°12'8''N 1°38'43''W | VAL | IA 2069 | Val (5) | 10.70 | 0.00 | 2.10 | 1.90 | Petroselinum |
| *Anacyclus clavatus* | 42°12'8''N 1°38'43''W | VAL | IA 2069 | Val (6) | 10.60 | 0.00 | 2.29 | 2.19 | Petroselinum |
| *Anacyclus clavatus* | 42°12'8''N 1°38'43''W | VAL | IA 2069 | Val (9) | 10.55 | 0.01 | 2.29 | 2.05 | Petroselinum |
| *Anacyclus clavatus*, *A. homogamos* | 32°43'1.9''N 5°47'56.5''W | OUA | AA 47 | AA47-9 | 9.73 | 0 | 1.89 | 2.64 | Petroselinum |
| *Anacyclus clavatus*, *A. homogamos* | 32°43'1.9''N 5°47'56.5''W | OUA | AA 47 | AA47-2 | 9.81 | 0.01 | 1.92 | 2.5 | Petroselinum |
| *Anacyclus clavatus*, *A. homogamos* | 32°43'1.9''N 5°47'56.5''W | OUA | AA 47 | AA47-7 | 9.8 | 0.02 | 2.35 | 2.81 | Petroselinum |
| *Anacyclus clavatus*, *A. homogamos* | 32°43'1.9''N 5°47'56.5''W | OUA | AA 47 | AA47-17 | 9.72 | 0.01 | 2.16 | 2.52 | Petroselinum |
| *Anacyclus clavatus*, *A. homogamos* | 32°43'1.9''N 5°47'56.5''W | OUA | AA 47 | AA47-3 | 9.62 | 0.01 | 2.4 | 2.46 | Petroselinum |
| *Anacyclus clavatus*, *A. radiatus* | 36°43'41.5''N 3°57'23.9''W | TORR | IA 2134 | Torr (2) | 10.60 | 0.00 | 1.93 | 1.78 | Petroselinum |
| *Anacyclus clavatus*, *A. radiatus* | 36°43'41.5''N 3°57'23.9''W | TORR | IA 2134 | Torr (3) | 9.39 | 0.01 | 1.89 | 2.20 | Petroselinum |
| *Anacyclus clavatus*, *A. radiatus* | 36°43'41.5''N 3°57'23.9''W | TORR | IA 2134 | Torr (8) | 9.59 | 0.00 | 1.94 | 1.87 | Petroselinum |
| *Anacyclus clavatus*, *A. radiatus* | 36°43'41.5''N 3°57'23.9''W | TORR | IA 2134 | Torr (11) | 9.66 | 0.00 | 2.05 | 1.89 | Petroselinum |
| *Anacyclus clavatus*, *A. radiatus* | 36°43'41.5''N 3°57'23.9''W | TORR | IA 2134 | Torr (12) | 9.66 | 0.00 | 2.09 | 2.02 | Petroselinum |
| *Anacyclus clavatus*, *A. valentinus* | 38°36'18.62''N 0°2'57.97''W | ALT | LM 4434-4435 | Alt34 (10) clavatoid | 10.47 | 0.32 | 2.89 | 2.64 | Petroselinum |
| *Anacyclus clavatus*, *A. valentinus* | 38°36'18.62''N 0°2'57.97''W | ALT | LM 4434-4435 | Alt34 (2) clavatoid | 9.64 | 0.01 | 2.59 | 2.34 | Petroselinum |
| *Anacyclus clavatus*, *A. valentinus* | 38°36'18.62''N 0°2'57.97''W | ALT | LM 4434-4435 | Alt34 (3) clavatoid | 10.65 | 0.03 | 2.51 | 2.23 | Petroselinum |
| *Anacyclus clavatus*, *A. valentinus* | 38°36'18.62''N 0°2'57.97''W | ALT | LM 4434-4435 | Alt34 (4) clavatoid | 10.54 | 0.08 | 2.14 | 2.00 | Petroselinum |
| *Anacyclus clavatus*, *A. valentinus* | 38°36'18.62''N 0°2'57.97''W | ALT | LM 4434-4435 | Alt34 (7) clavatoid | 10.55 | 0.03 | 2.76 | 2.59 | Petroselinum |
| *Anacyclus clavatus*, *A. valentinus* | 38°36'18.62''N 0°2'57.97''W | ALT | LM 4434-4435 | Alt35 (1) valentinoid | 9.68 | 0.01 | 2.67 | 2.51 | Petroselinum |
| *Anacyclus clavatus*, *A. valentinus* | 38°36'18.62''N 0°2'57.97''W | ALT | LM 4434-4435 | Alt35 (2) valentinoid | 8.73 | 0.04 | 2.74 | 2.72 | Petroselinum |
| *Anacyclus clavatus*, *A. valentinus* | 38°36'18.62''N 0°2'57.97''W | ALT | LM 4434-4435 | Alt35 (3) valentinoid | 10.30 | 0.02 | 2.24 | 1.92 | Petroselinum |
| *Anacyclus clavatus*, *A. valentinus* | 38°36'18.62''N 0°2'57.97''W | ALT | LM 4434-4435 | Alt35 (4) valentinoid | 9.12 | 0.01 | 2.96 | 2.46 | Petroselinum |
| *Anacyclus clavatus*, *A. valentinus* | 38°36'18.62''N 0°2'57.97''W | ALT | LM 4434-4435 | Alt35 (7) valentinoid | 11.55 | 0.00 | 2.53 | 2.06 | Petroselinum |
| *Anacyclus clavatus*, *A. valentinus* | 38°36'18.62''N 0°2'57.97''W | ALT | LM 4434-4435 | Alt35 (8) valentinoid | 9.48 | 0.00 | 3.26 | 2.82 | Petroselinum |
| *Anacyclus clavatus*, *A. valentinus* | 38°43'05.3"N 0°03'43.0"E | BEN | IA 2024 | Ben (1) | 9.73 | 0.00 | 2.37 | 1.98 | Petroselinum |
| *Anacyclus clavatus*, *A. valentinus* | 38°43'05.3"N 0°03'43.0"E | BEN | IA 2024 | Ben (10) | 9.71 | 0.01 | 3.52 | 3.41 | Petroselinum |
| *Anacyclus clavatus*, *A. valentinus* | 38°43'05.3"N 0°03'43.0"E | BEN | IA 2024 | Ben (2) | 9.72 | 0.02 | 2.81 | 2.67 | Petroselinum |
| *Anacyclus clavatus*, *A. valentinus* | 38°43'05.3"N 0°03'43.0"E | BEN | IA 2024 | Ben (3) | 9.78 | 0.01 | 2.90 | 2.89 | Petroselinum |
| *Anacyclus clavatus*, *A. valentinus* | 38°43'05.3"N 0°03'43.0"E | BEN | IA 2024 | Ben (6) | 9.82 | 0.01 | 2.32 | 2.82 | Petroselinum |
| *Anacyclus clavatus*, *A. valentinus* | 38°43'05.3"N 0°03'43.0"E | BEN | IA 2024 | Ben (7) | 9.68 | 0.01 | 2.64 | 2.51 | Petroselinum |
| *Anacyclus clavatus*, *A. valentinus* | 36°45'35''N 4°11'5.8''W | CAJ | IA 2136 | Caj (1) | 8.69 | 0.00 | 2.21 | 1.58 | Petroselinum |
| *Anacyclus clavatus*, *A. valentinus* | 36°45'35''N 4°11'5.8''W | CAJ | IA 2136 | Caj (10) | 8.63 | 0.01 | 1.94 | 2.07 | Petroselinum |
| *Anacyclus clavatus*, *A. valentinus* | 36°45'35''N 4°11'5.8''W | CAJ | IA 2136 | Caj (11) | 8.70 | 0.00 | 2.13 | 2.00 | Petroselinum |
| *Anacyclus clavatus*, *A. valentinus* | 36°45'35''N 4°11'5.8''W | CAJ | IA 2136 | Caj (3) | 9.12 | 0.01 | 2.01 | 1.90 | Petroselinum |
| *Anacyclus clavatus*, *A. valentinus* | 36°45'35''N 4°11'5.8''W | CAJ | IA 2136 | Caj (5) | 8.67 | 0.00 | 2.23 | 2.01 | Petroselinum |
| *Anacyclus clavatus*, *A. valentinus* | 36°45'35''N 4°11'5.8''W | CAJ | IA 2136 | Caj (6) | 8.61 | 0.00 | 1.92 | 1.80 | Petroselinum |
| *Anacyclus clavatus*, *A. valentinus* | 36°43'15.5''N 4°9'29.2''W | CHI | IA 2133 | Chi (1) | 8.58 | 0.00 | 2.48 | 2.17 | Petroselinum |
| *Anacyclus clavatus*, *A. valentinus* | 36°43'15.5''N 4°9'29.2''W | CHI | IA 2133 | Chi (11) | 8.62 | 0.01 | 2.46 | 2.22 | Petroselinum |
| *Anacyclus clavatus*, *A. valentinus* | 36°43'15.5''N 4°9'29.2''W | CHI | IA 2133 | Chi (2) | 8.66 | 0.00 | 2.29 | 2.31 | Petroselinum |
| *Anacyclus clavatus*, *A. valentinus* | 36°43'15.5''N 4°9'29.2''W | CHI | IA 2133 | Chi (4) | 8.77 | 0.00 | 1.94 | 1.93 | Petroselinum |
| *Anacyclus clavatus*, *A. valentinus* | 36°43'15.5''N 4°9'29.2''W | CHI | IA 2133 | Chi (5) | 8.67 | 0.00 | 2.50 | 2.52 | Petroselinum |
| *Anacyclus clavatus*, *A. valentinus* | 36°43'15.5''N 4°9'29.2''W | CHI | IA 2133 | Chi (8) | 8.74 | 0.01 | 2.03 | 1.89 | Petroselinum |
| *Anacyclus clavatus*, *A. valentinus* | 41°24'23''N 2°06'06''E | COL | OH 685-693 | OH 685 intermediate | 10.15 | 0.05 | 2.54 | 2.78 | Petroselinum |
| *Anacyclus clavatus*, *A. valentinus* | 41°24'23''N 2°06'06''E | COL | OH 685-693 | OH 686 intermediate | 10.31 | 0.05 | 2.22 | 2.10 | Petroselinum |
| *Anacyclus clavatus*, *A. valentinus* | 41°24'23''N 2°06'06''E | COL | OH 685-693 | OH 687 intermediate | 10.03 | 0.04 | 3.87 | 4.00 | Petroselinum |
| *Anacyclus clavatus*, *A. valentinus* | 41°24'23''N 2°06'06''E | COL | OH 685-693 | OH 688 valentinoid | 10.19 | 0.16 | 2.09 | 1.84 | Petroselinum |
| *Anacyclus clavatus*, *A. valentinus* | 41°24'23''N 2°06'06''E | COL | OH 685-693 | OH 689 valentinoid | 10.32 | 0.02 | 2.14 | 2.11 | Petroselinum |
| *Anacyclus clavatus*, *A. valentinus* | 41°24'23''N 2°06'06''E | COL | OH 685-693 | OH 690 valentinoid | 10.28 | 0.08 | 2.34 | 2.39 | Petroselinum |
| *Anacyclus clavatus*, *A. valentinus* | 41°24'23''N 2°06'06''E | COL | OH 685-693 | OH 691 clavatoid | 10.23 | 0.12 | 2.64 | 2.84 | Petroselinum |
| *Anacyclus clavatus*, *A. valentinus* | 41°24'23''N 2°06'06''E | COL | OH 685-693 | OH 692 clavatoid | 9.89 | 0.04 | 3.00 | 2.59 | Petroselinum |
| *Anacyclus clavatus*, *A. valentinus* | 41°24'23''N 2°06'06''E | COL | OH 685-693 | OH 693 clavatoid | 10.16 | 0.02 | 2.57 | 2.43 | Petroselinum |
| *Anacyclus clavatus*, *A. valentinus* | 42°32'1.61''N 2°37'21.74''W | ELC | IA 2065 | IA2065-12* | 10.33 | 0.01 | 2.12 | 2.7 | Petroselinum |
| *Anacyclus clavatus*, *A. valentinus* | 42°32'1.61''N 2°37'21.74''W | ELC | IA 2065 | IA2065-3 | 10.32 | 0.01 | 2.06 | 2.86 | Petroselinum |
| *Anacyclus clavatus*, *A. valentinus* | 42°32'1.61''N 2°37'21.74''W | ELC | IA 2065 | IA2065-5 | 10.45 | 0.01 | 2.15 | 2.89 | Petroselinum |
| *Anacyclus clavatus*, *A. valentinus* | 42°32'1.61''N 2°37'21.74''W | ELC | IA 2065 | IA2065-6 | 10.43 | 0.03 | 2.32 | 2.72 | Petroselinum |
| *Anacyclus clavatus*, *A. valentinus* | 42°32'1.61''N 2°37'21.74''W | ELC | IA 2065 | IA2065-7 | 10.69 | 0.01 | 2.1 | 2.96 | Petroselinum |
| *Anacyclus clavatus*, *A. valentinus* | 40°37'55.2''N 0°16'52''E | SEN | IA 2033-2034 | Sen33 (1) valentinoid | 9.53 | 0.01 | 2.16 | 2.10 | Petroselinum |
| *Anacyclus clavatus*, *A. valentinus* | 40°37'55.2''N 0°16'52''E | SEN | IA 2033-2034 | Sen33 (2) valentinoid | 10.38 | 0.01 | 2.05 | 1.68 | Petroselinum |
| *Anacyclus clavatus*, *A. valentinus* | 40°37'55.2''N 0°16'52''E | SEN | IA 2033-2034 | Sen33 (3) valentinoid | 10.57 | 0.01 | 2.10 | 2.10 | Petroselinum |
| *Anacyclus clavatus*, *A. valentinus* | 40°37'55.2''N 0°16'52''E | SEN | IA 2033-2034 | Sen33 (4) valentinoid | 10.25 | 0.01 | 1.92 | 1.84 | Petroselinum |
| *Anacyclus clavatus*, *A. valentinus* | 40°37'55.2''N 0°16'52''E | SEN | IA 2033-2034 | Sen33 (5) valentinoid | 9.11 | 0.00 | 2.06 | 1.87 | Petroselinum |
| *Anacyclus clavatus*, *A. valentinus* | 40°37'55.2''N 0°16'52''E | SEN | IA 2033-2034 | Sen33 (6) valentinoid | 8.41 | 0.01 | 2.10 | 2.33 | Petroselinum |
| *Anacyclus clavatus*, *A. valentinus* | 40°37'55.2''N 0°16'52''E | SEN | IA 2033-2034 | Sen34 (10) clavatoid | 10.41 | 0.00 | 2.45 | 2.20 | Petroselinum |
| *Anacyclus clavatus*, *A. valentinus* | 40°37'55.2''N 0°16'52''E | SEN | IA 2033-2034 | Sen34 (11) clavatoid | 10.31 | 0.00 | 2.11 | 1.82 | Petroselinum |
| *Anacyclus clavatus*, *A. valentinus* | 40°37'55.2''N 0°16'52''E | SEN | IA 2033-2034 | Sen34 (12) clavatoid | 10.17 | 0.02 | 2.36 | 2.27 | Petroselinum |
| *Anacyclus clavatus*, *A. valentinus* | 40°37'55.2''N 0°16'52''E | SEN | IA 2033-2034 | Sen34 (7) clavatoid | 10.40 | 0.01 | 2.31 | 2.43 | Petroselinum |
| *Anacyclus clavatus*, *A. valentinus* | 40°37'55.2''N 0°16'52''E | SEN | IA 2033-2034 | Sen34 (8) clavatoid | 10.68 | 0.00 | 2.09 | 1.89 | Petroselinum |
| *Anacyclus clavatus*, *A. valentinus* | 40°37'55.2''N 0°16'52''E | SEN | IA 2033-2034 | Sen34 (9) clavatoid | 10.54 | 0.01 | 2.48 | 2.37 | Petroselinum |
| *Anacyclus clavatus*, *A. valentinus* | 41°20'31.0"N 2°02'02.2"E | SBO | 25-IPL-1 | 25-IPL-1-1 clavatoid | 10.49 | 0.13 | 3.225 | 2.91 | Petroselinum |
| *Anacyclus clavatus*, *A. valentinus* | 41°20'31.0"N 2°02'02.2"E | SBO | 25-IPL-1 | 25-IPL-1-2 clavatoid | 9.46 | 0.05 | 3.12 | 2.98 | Petroselinum |
| *Anacyclus clavatus*, *A. valentinus* | 41°20'31.0"N 2°02'02.2"E | SBO | 25-IPL-1 | 25-IPL-1-3 clavatoid | 10.49 | 0.12 | 3.64 | 3.6 | Petroselinum |
| *Anacyclus clavatus*, *A. valentinus* | 41°20'31.0"N 2°02'02.2"E | SBO | 25-IPL-1 | 25-IPL-1-4 clavatoid | 10.42 | 0.12 | 3.635 | 3.55 | Petroselinum |
| *Anacyclus clavatus*, *A. valentinus* | 41°20'31.0"N 2°02'02.2"E | SBO | 25-IPL-1 | 25-IPL-1-5 clavatoid | 10.37 | 0.04 | 3.635 | 3.43 | Petroselinum |
| *Anacyclus clavatus*, *A. valentinus* | 41°20'31.0"N 2°02'02.2"E | SBO | 25-IPL-1 | 25-IPL-1-6 clavatoid | 10.33 | 0.05 | 3.635 | 3.47 | Petroselinum |
| *Anacyclus clavatus*, *A. valentinus* | 41°20'31.0"N 2°02'02.2"E | SBO | 25-IPL-1 | 25-IPL-1-7 clavatoid | 10.31 | 0.15 | 3.205 | 2.96 | Petroselinum |
| *Anacyclus clavatus*, *A. valentinus* | 41°20'31.0"N 2°02'02.2"E | SBO | 25-IPL-1 | 25-IPL-1-8 clavatoid | 10.28 | 0 | 3.125 | 3.31 | Petroselinum |
| *Anacyclus clavatus*, *A. valentinus* | 41°20'31.0"N 2°02'02.2"E | SBO | 25-IPL-1 | 25-IPL-1-9 clavatoid | 9.72 | 0.06 | 3.25 | 3.23 | Petroselinum |
| *Anacyclus clavatus*, *A. valentinus* | 41°20'31.0"N 2°02'02.2"E | SBO | 25-IPL-1 | 25-IPL-1-10 clavatoid | 10.16 | 0.06 | 2.76 | 2.63 | Petroselinum |
| *Anacyclus clavatus*, *A. valentinus* | 41°20'31.0"N 2°02'02.2"E | SBO | 25-IPL-2 | 25-IPL-2-1 intermediate | 8.98 | 0.1 | 2.615 | 2.4 | Petroselinum |
| *Anacyclus clavatus*, *A. valentinus* | 41°20'31.0"N 2°02'02.2"E | SBO | 25-IPL-2 | 25-IPL-2-2 intermediate | 9.2 | 0.05 | 2.64 | 2.7 | Petroselinum |
| *Anacyclus clavatus*, *A. valentinus* | 41°20'31.0"N 2°02'02.2"E | SBO | 25-IPL-2 | 25-IPL-2-3 intermediate | 9.17 | 0.08 | 2.81 | 2.7 | Petroselinum |
| *Anacyclus clavatus*, *A. valentinus* | 41°20'31.0"N 2°02'02.2"E | SBO | 25-IPL-2 | 25-IPL-2-4 intermediate | 9.35 | 0.08 | 2.675 | 2.69 | Petroselinum |
| *Anacyclus clavatus*, *A. valentinus* | 41°20'31.0"N 2°02'02.2"E | SBO | 25-IPL-2 | 25-IPL-2-5 intermediate | 9.56 | 0.06 | 2.985 | 2.68 | Petroselinum |
| *Anacyclus clavatus*, *A. valentinus* | 41°20'31.0"N 2°02'02.2"E | SBO | 25-IPL-2 | 25-IPL-2-6 intermediate | 9.2 | 0.04 | 2.8 | 2.77 | Petroselinum |
| *Anacyclus clavatus*, *A. valentinus* | 41°20'31.0"N 2°02'02.2"E | SBO | 25-IPL-2 | 25-IPL-2-7 intermediate | 9.14 | 0.03 | 2.885 | 2.7 | Petroselinum |
| *Anacyclus clavatus*, *A. valentinus* | 41°20'31.0"N 2°02'02.2"E | SBO | 25-IPL-2 | 25-IPL-2-8 intermediate | 9.29 | 0.05 | 2.995 | 2.78 | Petroselinum |
| *Anacyclus clavatus*, *A. valentinus* | 41°20'31.0"N 2°02'02.2"E | SBO | 25-IPL-2 | 25-IPL-2-9 intermediate | 9.24 | 0.05 | 2.63 | 2.66 | Petroselinum |
| *Anacyclus clavatus*, *A. valentinus* | 41°20'31.0"N 2°02'02.2"E | SBO | 25-IPL-2 | 25-IPL-2-10 intermediate | 9.18 | 0.06 | 2.835 | 2.63 | Petroselinum |
| *Anacyclus clavatus*, *A. valentinus* | 41°20'31.0"N 2°02'02.2"E | SBO | 25-IPL-3 | 25-IPL-3-1 valentinoid | 9.38 | 0.03 | 2.91 | 2.91 | Petroselinum |
| *Anacyclus clavatus*, *A. valentinus* | 41°20'31.0"N 2°02'02.2"E | SBO | 25-IPL-3 | 25-IPL-3-2 valentinoid | 9.31 | 0.03 | 3.175 | 2.97 | Petroselinum |
| *Anacyclus clavatus*, *A. valentinus* | 41°20'31.0"N 2°02'02.2"E | SBO | 25-IPL-3 | 25-IPL-3-3 valentinoid | 8.18 | 0.02 | 2.835 | 2.61 | Petroselinum |
| *Anacyclus clavatus*, *A. valentinus* | 41°20'31.0"N 2°02'02.2"E | SBO | 25-IPL-3 | 25-IPL-3-4 valentinoid | 8.89 | 0.06 | 3.005 | 2.87 | Petroselinum |
| *Anacyclus clavatus*, *A. valentinus* | 41°20'31.0"N 2°02'02.2"E | SBO | 25-IPL-3 | 25-IPL-3-5 valentinoid | 10.17 | 0.07 | 2.91 | 2.84 | Petroselinum |
| *Anacyclus clavatus*, *A. valentinus* | 41°20'31.0"N 2°02'02.2"E | SBO | 25-IPL-3 | 25-IPL-3-6 valentinoid | 10.25 | 0.11 | 2.975 | 2.84 | Petroselinum |
| *Anacyclus clavatus*, *A. valentinus* | 41°20'31.0"N 2°02'02.2"E | SBO | 25-IPL-3 | 25-IPL-3-7 valentinoid | 8.08 | 0.06 | 2.86 | 2.79 | Petroselinum |
| *Anacyclus clavatus*, *A. valentinus* | 41°20'31.0"N 2°02'02.2"E | SBO | 25-IPL-3 | 25-IPL-3-8 valentinoid | 8.2 | 0.05 | 3.045 | 2.95 | Petroselinum |
| *Anacyclus clavatus*, *A. valentinus* | 41°20'31.0"N 2°02'02.2"E | SBO | 25-IPL-3 | 25-IPL-3-9 valentinoid | 8.17 | 0.03 | 2.6 | 2.58 | Petroselinum |
| *Anacyclus clavatus*, *A. valentinus* | 41°20'31.0"N 2°02'02.2"E | SBO | 25-IPL-3 | 25-IPL-3-10 valentinoid | 8.19 | 0.04 | 2.71 | 2.49 | Petroselinum |
| *Anacyclus homogamos* | 30°44'2.9''N 7°47'51.2''W | ASK | AQ 3505 | AQ3505 | 10.6 | 0.03 | 1.72 | 1.93 | Petroselinum |
| *Anacyclus homogamos* | 31°15'4''N 7°58'40''W | ASN | IA 2115 | IA2115-11 | 10.6 | 0.02 | 2.58 | 3.12 | Petroselinum |
| *Anacyclus homogamos* | 31°15'4''N 7°58'40''W | ASN | IA 2115 | IA2115-3 | 10.85 | 0 | 2.33 | 2.77 | Petroselinum |
| *Anacyclus homogamos* | 31°15'4''N 7°58'40''W | ASN | IA 2115 | IA2115-4 | 10.79 | 0.01 | 2.07 | 2.79 | Petroselinum |
| *Anacyclus homogamos* | 31°15'4''N 7°58'40''W | ASN | IA 2115 | IA2115-7 | 10.76 | 0.01 | 2.26 | 2.88 | Petroselinum |
| *Anacyclus homogamos* | 31°19'55''N 7°24'32''W | IMO | RG 1275 | RG1275-20 | 10.2 | 0.02 | 2.41 | 2.69 | Petroselinum |
| *Anacyclus homogamos* | 31°19'55''N 7°24'32''W | IMO | RG 1275 | RG1275-2 | 10.52 | 0.01 | 2.35 | 2.56 | Petroselinum |
| *Anacyclus homogamos* | 31°19'55''N 7°24'32''W | IMO | RG 1275 | RG1275-1 | 10.32 | 0.03 | 2.4 | 2.5 | Petroselinum |
| *Anacyclus homogamos* | 31°19'55''N 7°24'32''W | IMO | RG 1275 | RG1275-12 | 10.55 | 0.02 | 2.25 | 2.6 | Petroselinum |
| *Anacyclus homogamos* | 31°19'55''N 7°24'32''W | IMO | RG 1275 | RG1275-15 | 10.36 | 0 | 2.44 | 2.71 | Petroselinum |
| *Anacyclus homogamos*, *A. valentinus* | 32°35'55.3"N 6°38'44.0"W | KSI | AA 45 | Ksi (1) | 10.00 | 0.01 | 1.90 | 1.74 | Petroselinum |
| *Anacyclus homogamos*, *A. valentinus* | 32°35'55.3"N 6°38'44.0"W | KSI | AA 45 | Ksi (3) | 9.80 | 0.00 | 1.94 | 1.73 | Petroselinum |
| *Anacyclus homogamos*, *A. valentinus* | 32°35'55.3"N 6°38'44.0"W | KSI | AA 45 | Ksi (4) | 9.88 | 0.00 | 2.05 | 1.49 | Petroselinum |
| *Anacyclus homogamos*, *A. valentinus* | 32°35'55.3"N 6°38'44.0"W | KSI | AA 45 | Ksi (8) | 9.89 | 0.01 | 1.96 | 1.85 | Petroselinum |
| *Anacyclus homogamos*, *A. valentinus* | 32°35'55.3"N 6°38'44.0"W | KSI | AA 45 | Ksi (12) | 9.97 | 0.01 | 2.12 | 1.73 | Petroselinum |
| *Anacyclus linearilobus* | 35°45'0.32’''N 0°49'50.52''W | BMO | IA 2340 | IA 2340 (2)-1 | 13.65 | 0.01 | 2.11 | 1.92 | Petroselinum |
| *Anacyclus linearilobus* | 35°45'0.32’''N 0°49'50.52''W | BMO | IA 2340 | IA 2340 (2)-2 | 13.50 | 0.01 | 2.00 | 1.96 | Petroselinum |
| *Anacyclus linearilobus* | 35°45'0.32’''N 0°49'50.52''W | BMO | IA 2340 | IA 2340 (2)-3 | 13.21 | 0.04 | 2.23 | 2.05 | Petroselinum |
| *Anacyclus linearilobus* | 35°45'0.32’''N 0°49'50.52''W | BMO | IA 2340 | IA 2340 (5)-1 | 13.37 | 0.05 | 2.40 | 2.20 | Petroselinum |
| *Anacyclus linearilobus* | 35°45'0.32’''N 0°49'50.52''W | BMO | IA 2340 | IA 2340 (5)-2 | 13.31 | 0.02 | 1.86 | 1.67 | Petroselinum |
| *Anacyclus linearilobus* | 35°44'40.1"N 0°50'03.0"W | BOM | IA 2338 | IA-2338(1) | 13.42 | 0.04 | 2.01 | 1.64 | Petroselinum |
| *Anacyclus linearilobus* | 35°44'40.1"N 0°50'03.0"W | BOM | IA 2338 | IA-2338(2) | 13.30 | 0.02 | 2.07 | 1.60 | Petroselinum |
| *Anacyclus linearilobus* | 35°44'40.1"N 0°50'03.0"W | BOM | IA 2338 | IA-2338(3) | 13.39 | 0.06 | 2.33 | 2.04 | Petroselinum |
| *Anacyclus linearilobus* | 35°44'40.1"N 0°50'03.0"W | BOM | IA 2338 | IA-2338(4) | 13.43 | 0.09 | 2.28 | 1.68 | Petroselinum |
| *Anacyclus linearilobus* | 35°44'40.1"N 0°50'03.0"W | BOM | IA 2338 | IA-2338(5) | 13.40 | 0.05 | 2.03 | 1.71 | Petroselinum |
| *Anacyclus linearilobus* | 35°44'40.1"N 0°50'03.0"W | BOM | IA 2338 | IA-2338(6) | 13.33 | 0.02 | 2.15 | 1.80 | Petroselinum |
| *Anacyclus linearilobus* | 35°44'40.1"N 0°50'03.0"W | BOM | IA 2338 | IA-2338(7) | 13.49 | 0.05 | 1.98 | 2.19 | Petroselinum |
| *Anacyclus linearilobus* | 35°44'40.1"N 0°50'03.0"W | BOM | IA 2338 | IA-2338(8) | 13.34 | 0.06 | 2.25 | 1.73 | Petroselinum |
| *Anacyclus linearilobus* | 35°44'40.1"N 0°50'03.0"W | BOM | IA 2338 | IA-2338(9) | 13.50 | 0.06 | 1.99 | 1.72 | Petroselinum |
| *Anacyclus linearilobus* | 35°44'40.1"N 0°50'03.0"W | BOM | IA 2338 | IA-2338(10) | 13.47 | 0.04 | 2.06 | 1.93 | Petroselinum |
| *Anacyclus linearilobus* | 35°44'40.1"N 0°50'03.0"W | BOM | IA 2338 | IA-2338(11) | 13.60 | 0.05 | 1.95 | 1.90 | Petroselinum |
| *Anacyclus linearilobus* | 35°44'40.1"N 0°50'03.0"W | BOM | IA 2338 | IA-2338(12) | 13.66 | 0.02 | 2.09 | 1.90 | Petroselinum |
| *Anacyclus linearilobus* | 35°47'22.65''N 0°9'9.84''W | MAC | IA 2345 | IA 2345 (1)-1 | 13.73 | 0.02 | 1.70 | 1.84 | Petroselinum |
| *Anacyclus linearilobus* | 35°47'22.65''N 0°9'9.84''W | MAC | IA 2345 | IA 2345 (1)-2 | 13.95 | 0.01 | 2.17 | 1.80 | Petroselinum |
| *Anacyclus linearilobus* | 35°47'22.65''N 0°9'9.84''W | MAC | IA 2345 | IA 2345 (6)-1 | 13.95 | 0.00 | 1.91 | 1.80 | Petroselinum |
| *Anacyclus linearilobus* | 35°47'22.65''N 0°9'9.84''W | MAC | IA 2345 | IA 2345 (6)-2 | 13.64 | 0.01 | 1.81 | 1.82 | Petroselinum |
| *Anacyclus linearilobus* | 35°47'22.65''N 0°9'9.84''W | MAC | IA 2345 | IA 2345 (6)-3 | 13.88 | 0.03 | 2.23 | 2.46 | Petroselinum |
| *Anacyclus linearilobus* | 35°47'22.65''N 0°9'9.84''W | MAC | IA 2345 | IA 2345 (6)-4 | 13.81 | 0.03 | 1.90 | 2.03 | Petroselinum |
| *Anacyclus linearilobus* | 35°45'43.48''N 0°49'46.68''W | MOB | IA 2339 | IA 2339 (3)-1 | 13.45 | 0.06 | 3.21 | 2.78 | Petroselinum |
| *Anacyclus linearilobus* | 35°45'43.48''N 0°49'46.68''W | MOB | IA 2339 | IA 2339 (3)-2 | 13.52 | 0.00 | 2.83 | 2.53 | Petroselinum |
| *Anacyclus linearilobus* | 35°45'43.48''N 0°49'46.68''W | MOB | IA 2339 | IA 2339 (3)-3 | 13.32 | 0.06 | 2.76 | 2.14 | Petroselinum |
| *Anacyclus maroccanus* | 31°53'31''N 7°26'23''W | MAR | AA 33 | AA33-3 | 9.9 | 0.02 | 2.4 | 2.76 | Petroselinum |
| *Anacyclus maroccanus* | 31°53'31''N 7°26'23''W | MAR | AA 33 | AA33-4 | 9.79 | 0.03 | 2.26 | 2.79 | Petroselinum |
| *Anacyclus maroccanus* | 31°53'31''N 7°26'23''W | MAR | AA 33 | AA33-8 | 9.69 | 0.04 | 2.3 | 2.95 | Petroselinum |
| *Anacyclus maroccanus* | 31°53'31''N 7°26'23''W | MAR | AA 33 | AA33-5 | 9.89 | 0.01 | 2.16 | 2.93 | Petroselinum |
| *Anacyclus maroccanus* | 31°53'31''N 7°26'23''W | MAR | AA 33 | AA33-11 | 9.91 | 0.01 | 2.15 | 2.8 | Petroselinum |
| *Anacyclus maroccanus* | 31°35'16.86''N 9°4'17.52''W | SID | IA 2099 | IA2099-3 | 9.72 | 0.02 | 2.1 | 2.25 | Petroselinum |
| *Anacyclus maroccanus* | 31°35'16.86''N 9°4'17.52''W | SID | IA 2099 | IA2099-4 | 9.8 | 0.03 | 2.25 | 2.22 | Petroselinum |
| *Anacyclus maroccanus* | 31°35'16.86''N 9°4'17.52''W | SID | IA 2099 | IA2099-8 | 9.81 | 0.03 | 2.35 | 2.2 | Petroselinum |
| *Anacyclus maroccanus* | 31°35'16.86''N 9°4'17.52''W | SID | IA 2099 | IA2099-6 | 9.65 | 0.01 | 2.35 | 2.42 | Petroselinum |
| *Anacyclus maroccanus* | 31°37'55.66''N 8°15'10.67''W | TNI | IA 2097 | IA2097-Valencia | 9.84 | 0.01 | 2.16 | 2.03 | Petroselinum |
| *Anacyclus maroccanus* | 31°37'55.66''N 8°15'10.67''W | TNI | IA 2097 | IA2097-4 | 9.82 | 0.01 | 2.1 | 2.25 | Petroselinum |
| *Anacyclus monanthos* | 33°53'11''N 10°1'6.2''E | GAB | CA 16194 | CA16194-1 | 11.83 | 0.01 | 2.04 | 1.86 | Petroselinum |
| *Anacyclus monanthos* | 33°44'33''N 10°0'56''E | MAT | CA 16233 | CA162333-Valencia | 11.83 | 0.04 | 2.55 | 2.88 | Petroselinum |
| *Anacyclus pyrethrum* | 30°44'37.4''N 7°42'24.8''W | ANS | AQ 3531 | AQ3531-7 | 13.44 | 0.01 | 2.9 | 3.06 | Petroselinum |
| *Anacyclus pyrethrum* | 30°44'37.4''N 7°42'24.8''W | ANS | AQ 3531 | AQ3531-9* | 13.43 | 0.05 | 2.46 | 3.45 | Petroselinum |
| *Anacyclus pyrethrum* | 30°44'37.4''N 7°42'24.8''W | ANS | AQ 3531 | AQ3531-11 | 13.37 | 0.04 | 2.46 | 3.23 | Petroselinum |
| *Anacyclus pyrethrum* | 30°44'37.4''N 7°42'24.8''W | ANS | AQ 3531 | AQ3531-8 | 13.4 | 0.04 | 2.12 | 3.19 | Petroselinum |
| *Anacyclus pyrethrum* | 30°44'37.4''N 7°42'24.8''W | ANS | AQ 3531 | AQ3531-3 | 13.32 | 0.02 | 2.4 | 3.19 | Petroselinum |
| *Anacyclus pyrethrum* | 38°38'25''N 2°22'41.26’''W | PEN | IA 2334 | IA2334-10 | 12.71 | 0.03 | 2.67 | 2.93 | Petroselinum |
| Anacyclus *radiatus* ssp. *coronarius* | 31°32'1.55''N 9°44'37.83''W | ESS | IA 2105 | IA2105-5 | 12.28 | 0.03 | 2.17 | 3.32 | Petroselinum |
| Anacyclus *radiatus* ssp. *coronarius* | 31°32'1.55''N 9°44'37.83''W | ESS | IA 2105 | IA2105-1 | 12.64 | 0 | 2.52 | 3.27 | Petroselinum |
| Anacyclus *radiatus* ssp. *coronarius* | 31°32'1.55''N 9°44'37.83''W | ESS | IA 2105 | IA2105-4 | 12.72 | 0.01 | 1.86 | 1.6 | Petroselinum |
| Anacyclus *radiatus* ssp. *coronarius* | 31°32'1.55''N 9°44'37.83''W | ESS | IA 2105 | IA2105-3 | 12.54 | 0.02 | 2 | 2.23 | Petroselinum |
| Anacyclus *radiatus* ssp. *coronarius* | 31°32'1.55''N 9°44'37.83''W | ESS | IA 2105 | IA2105-2 | 12.69 | 0.03 | 2.1 | 2.15 | Petroselinum |
| *Anacyclus radiatus* ssp. *radiatus* | 39°5'42''N 6°0'51''W | BUR | IA 2177 | IA2177-1 | 12.44 | 0.02 | 2.72 | 2.96 | Petroselinum |
| *Anacyclus radiatus* ssp. *radiatus* | 39°5'42''N 6°0'51''W | BUR | IA 2177 | IA2177-4 | 12.43 | 0.02 | 2.75 | 2.74 | Petroselinum |
| *Anacyclus radiatus* ssp. *radiatus* | 39°5'42''N 6°0'51''W | BUR | IA 2177 | IA2177-13 | 12.61 | 0.01 | 2.86 | 3.03 | Petroselinum |
| *Anacyclus radiatus* ssp. *radiatus* | 39°5'42''N 6°0'51''W | BUR | IA 2177 | IA2177-14 | 12.54 | 0.01 | 2.37 | 2.43 | Petroselinum |
| *Anacyclus radiatus* ssp. *radiatus* | 39°5'42''N 6°0'51''W | BUR | IA 2177 | IA2177-16 | 12.94 | 0.01 | 2.66 | 2.64 | Petroselinum |
| *Anacyclus radiatus* ssp. *radiatus* | 35°20'15.9''N 5°21'46.5''W | CHE | IA 2180 | IA2180-5 | 12.22 | 0.01 | 3.03 | 3.04 | Petroselinum |
| *Anacyclus radiatus* ssp. *radiatus* | 35°20'15.9''N 5°21'46.5''W | CHE | IA 2180 | IA2180-6 | 12.49 | 0.01 | 2.45 | 2.81 | Petroselinum |
| *Anacyclus radiatus* ssp. *radiatus* | 35°20'15.9''N 5°21'46.5''W | CHE | IA 2180 | IA2180-7 | 12.35 | 0.05 | 2.47 | 2.83 | Petroselinum |
| *Anacyclus radiatus* ssp. *radiatus* | 35°20'15.9''N 5°21'46.5''W | CHE | IA 2180 | IA2180-4 | 12.56 | 0.02 | 2.56 | 3.08 | Petroselinum |
| *Anacyclus radiatus* ssp. *radiatus* | 36°33'6.5''N 4°41'47.3''W | ENT | IA 2138 | IA2138-4 | 12.47 | 0.04 | 2.64 | 3.23 | Petroselinum |
| *Anacyclus radiatus* ssp. *radiatus* | 36°33'6.5''N 4°41'47.3''W | ENT | IA 2138 | IA2138-8 | 12.48 | 0.01 | 2.62 | 3.3 | Petroselinum |
| *Anacyclus radiatus* ssp. *radiatus* | 36°33'6.5''N 4°41'47.3''W | ENT | IA 2138 | IA2138-10 | 12.65 | 0.01 | 2.45 | 2.87 | Petroselinum |
| *Anacyclus radiatus* ssp. *radiatus* | 36°33'6.5''N 4°41'47.3''W | ENT | IA 2138 | IA2138-12 | 12.32 | 0.03 | 2.54 | 2.74 | Petroselinum |
| *Anacyclus radiatus* ssp. *radiatus* | 36°33'6.5''N 4°41'47.3''W | ENT | IA 2138 | IA2138-5 | 12.47 | 0.03 | 2.64 | 3.23 | Petroselinum |
| *Anacyclus valentinus* | 40°56’'6.5''N 0°51'24.4''E | ALM | IA 2038 | IA2038 | 8.38 | 0.01 | 2.21 | 2.48 | Petroselinum |
| *Anacyclus valentinus* | 42°46'10.5"N 3°02'15.7"E | ARG | OH 1058 | OH 1058-1 | 8.39 | 0.01 | 2.84 | 2.88 | Petroselinum |
| *Anacyclus valentinus* | 42°46'10.5"N 3°02'15.7"E | ARG | OH 1058 | OH 1058-2 | 8.43 | 0.03 | 3.02 | 3.12 | Petroselinum |
| *Anacyclus valentinus* | 42°46'10.5"N 3°02'15.7"E | ARG | OH 1058 | OH 1058-3 | 8.30 | 0.02 | 3.30 | 3.40 | Petroselinum |
| *Anacyclus valentinus* | 42°46'10.5"N 3°02'15.7"E | ARG | OH 1058 | OH 1058-4 | 8.37 | 0.02 | 2.48 | 2.69 | Petroselinum |
| *Anacyclus valentinus* | 42°46'10.5"N 3°02'15.7"E | ARG | OH 1058 | OH 1058-5 | 8.37 | 0.04 | 3.46 | 3.62 | Petroselinum |
| *Anacyclus valentinus* | 41°38'26.2"N 2°10'23.1"E | CAL | GR 560 | IPL [8-VI-25]1 | 8.33 | 0.03 | 2.68 | 2.79 | Petroselinum |
| *Anacyclus valentinus* | 41°38'26.2"N 2°10'23.1"E | CAL | GR 560 | IPL [8-VI-25]2 | 8.29 | 0.00 | 2.46 | 2.58 | Petroselinum |
| *Anacyclus valentinus* | 41°38'26.2"N 2°10'23.1"E | CAL | GR 560 | IPL [8-VI-25]3 | 8.44 | 0.00 | 2.49 | 2.60 | Petroselinum |
| *Anacyclus valentinus* | 41°38'26.2"N 2°10'23.1"E | CAL | GR 560 | IPL [8-VI-25]4 | 8.33 | 0.01 | 2.45 | 2.41 | Petroselinum |
| *Anacyclus valentinus* | 41°38'26.2"N 2°10'23.1"E | CAL | GR 560 | IPL [8-VI-25]5 | 8.32 | 0.01 | 2.21 | 2.18 | Petroselinum |
| *Anacyclus valentinus* | 41°38'26.2"N 2°10'23.1"E | CAL | GR 560 | IPL [8-VI-25]6 | 8.31 | 0.01 | 2.25 | 2.39 | Petroselinum |
| *Anacyclus valentinus* | 41°38'26.2"N 2°10'23.1"E | CAL | GR 560 | IPL [8-VI-25]7 | 8.33 | 0 | 2.47 | 2.7 | Petroselinum |
| *Anacyclus valentinus* | 41°38'26.2"N 2°10'23.1"E | CAL | GR 560 | IPL [8-VI-25]8 | 8.28 | 0.02 | 2.50 | 2.71 | Petroselinum |
| *Anacyclus valentinus* | 41°38'26.2"N 2°10'23.1"E | CAL | GR 560 | IPL [8-VI-25]9 | 8.3 | 0 | 2.43 | 2.46 | Petroselinum |
| *Anacyclus valentinus* | 41°38'26.2"N 2°10'23.1"E | CAL | GR 560 | IPL [8-VI-25]10 | 8.41 | 0 | 2.51 | 2.63 | Petroselinum |
| *Anacyclus valentinus* | 42°15'47.2''N 3°7'45.5''E | CAS | IA 2059 | IA2059-1 | 8.36 | 0 | 2.44 | 3.25 | Petroselinum |
| *Anacyclus valentinus* | 42°15'47.2''N 3°7'45.5''E | CAS | IA 2059 | IA2059-2 | 8.64 | 0 | 2.21 | 2.98 | Petroselinum |
| *Anacyclus valentinus* | 42°15'47.2''N 3°7'45.5''E | CAS | IA 2059 | IA2059-3 | 8.34 | 0.02 | 2.37 | 2.51 | Petroselinum |
| *Anacyclus valentinus* | 42°15'47.2''N 3°7'45.5''E | CAS | IA 2059 | IA2059-4* | 8.32 | 0 | 2.52 | 2.9 | Petroselinum |
| *Anacyclus valentinus* | 41°21'44''N 2°09'34''E | MON | OH (V-2017) | OH s.n. (V-2017)/1 | 8.33 | 0.01 | 2.42 | 2.44 | Petroselinum |
| *Anacyclus valentinus* | 41°21'44''N 2°09'34''E | MON | OH (V-2017) | OH s.n. (V-2017)/2 | 8.32 | 0.03 | 2.66 | 2.57 | Petroselinum |
| *Anacyclus valentinus* | 41°21'44''N 2°09'34''E | MON | OH (V-2017) | OH s.n. (V-2017)/3 | 8.39 | 0.12 | 2.40 | 2.35 | Petroselinum |
| *Anacyclus valentinus* | 41°21'44''N 2°09'34''E | MON | OH 1057 | OH 1057-1 | 8.19 | 0.07 | 2.97 | 3.17 | Petroselinum |
| *Anacyclus valentinus* | 41°21'44''N 2°09'34''E | MON | OH 1057 | OH 1057-2 | 8.21 | 0.04 | 2.76 | 2.72 | Petroselinum |
| *Anacyclus valentinus* | 41°21'44''N 2°09'34''E | MON | OH 1057 | OH 1057-3 | 8.15 | 0.03 | 2.85 | 2.77 | Petroselinum |
| *Anacyclus valentinus* | 41°21'44''N 2°09'34''E | MON | OH 1057 | OH 1057-4 | 8.27 | 0.01 | 3.35 | 3.32 | Petroselinum |
| *Anacyclus valentinus* | 41°21'44''N 2°09'34''E | MON | OH 1057 | OH 1057-5 | 7.95 | 0.05 | 3.15 | 2.93 | Petroselinum |
| *Anacyclus valentinus* | 41°21'44''N 2°09'34''E | MON | OH 1057 | OH 1057-6 | 8.21 | 0.07 | 3.62 | 3.23 | Petroselinum |
| *Anacyclus valentinus* | 41°21'44''N 2°09'34''E | MON | OH 1057 | OH 1057-7 | 8.32 | 0.05 | 3.21 | 3.33 | Petroselinum |
| *Anacyclus valentinus* | 41°21'44''N 2°09'34''E | MON | OH 1057 | OH 1057-8 | 7.99 | 0.08 | 3.49 | 3.47 | Petroselinum |
| *Anacyclus valentinus* | 41°21'44''N 2°09'34''E | MON | OH 1057 | OH 1057-9 | 8.01 | 0.02 | 3.75 | 3.77 | Petroselinum |
| *Anacyclus valentinus* | 41°21'44''N 2°09'34''E | MON | OH 1057 | OH 1057-10 | 8.1 | 0.03 | 3.11 | 3.18 | Petroselinum |
| *Anacyclus valentinus* | 41°21'44''N 2°09'34''E | MON | OH 1057 | OH 1057-11 | 8.09 | 0.02 | 3.21 | 3.21 | Petroselinum |
| *Anacyclus valentinus* | 34°57'50.6"N 4°24'2.1"W | TAR | MS 1072 | MS1072-1 | 9.18 | 0.03 | 2.52 | 2.98 | Petroselinum |
| *Anacyclus valentinus* | 34°57'50.6"N 4°24'2.1"W | TAR | MS 1072 | MS1072-2 | 9.15 | 0.01 | 2.22 | 2.94 | Petroselinum |
| *Anacyclus valentinus* | 34°57'50.6"N 4°24'2.1"W | TAR | MS 1072 | MS1072-3* | 9.21 | 0.01 | 2.38 | 3.13 | Petroselinum |
| *Cota nigellifolia* | Iran: Kermanshah, Bisoton |  | Ker | Ker (1) | 18.25 | 0.06 | 2.12 | 1.62 | Pisum |
| *Cota nigellifolia* | Iran: Kermanshah, Bisoton |  | Ker | Ker (2) | 18.38 | 0.24 | 1.84 | 2.13 | Pisum |
| *Cota nigellifolia* | Iran: Kermanshah, Bisoton |  | Ker | Ker (3) | 18.46 | 0.05 | 1.83 | 1.62 | Pisum |
| *Cota nigellifolia* | Iran: Kermanshah, Bisoton |  | Ker | Ker (4) | 18.44 | 0.10 | 1.76 | 1.69 | Pisum |
| *Cota nigellifolia* | Iran: Kermanshah, Bisoton |  | Ker | Ker (5) | 18.30 | 0.07 | 1.88 | 1.66 | Pisum |
| *Cota nigellifolia* | Israel: Mount Hermon |  | 101336 | 101336-11 | 18.96 | 0.02 | 2.02 | 1.99 | Petroselinum |
| *Cota nigellifolia* | Israel: Mount Hermon |  | 101336 | 101336-2 | 18.85 | 0.05 | 2.56 | 2.2 | Petroselinum |
| *Cota nigellifolia* | Israel: Mount Hermon |  | 101336 | 101336-3 | 18.87 | 0.04 | 2.84 | 2.2 | Petroselinum |
| *Cota nigellifolia* | Israel: Mount Hermon |  | 101336 | 101336-4 | 18.71 | 0.05 | 2.52 | 1.8 | Petroselinum |
| *Cota nigellifolia* | Israel: Mount Hermon |  | 101336 | 101336-5 | 18.65 | 0.03 | 2.13 | 1.82 | Petroselinum |
