# Supplementary material for: Inside daisies’ evolutionary tangle: new insights into the hybridogenic genus Anacyclus (Asteraceae, Anthemideae)
Source: PeerJ. 2026 Aug 27;14:e21638. doi: 10.7717/peerj.21638 (PMC13528631; doi:10.7717/peerj.21638)
Supplement: Supplementary file 8 [file peerj-14-21638-s011.docx]

**Table S3.** Details of the samples used in the population genetic analyses.

| Code | Code POP | Code ind | Species | Latitude | Longitude | Alt (m) | date | collection number | ITS | cp DNA | ribotype | haplotype | ITS Genbank codes | rpL32F-trnL Genbank codes | ycf1-rps15 Genbank codes |
| --- | --- | --- | --- | --- | --- | --- | --- | --- | --- | --- | --- | --- | --- | --- | --- |
| AA1_clav_517_10 | CAR | CAR1 | *A. clavatus* | 36.69694444 | -3.459166667 | 13 | 27-IV-2011 | Agudo 1 | yes | yes | III | H | PX447960 | PX505132 | PX505207 |
| AA1_clav_Car2 | CAR | CAR2 | *A. clavatus* | 36.69694444 | -3.459166667 | 13 | 27-IV-2011 | Agudo 1 | yes | no | III | na | PX447963 | na | na |
| AA1_clav_Car3 | CAR | CAR3 | *A. clavatus* | 36.69694444 | -3.459166667 | 13 | 27-IV-2011 | Agudo 1 | yes | yes | III | A | PX447961 | PX505134 | PX505209 |
| AA1_clav_Car4 | CAR | CAR4 | *A. clavatus* | 36.69694444 | -3.459166667 | 13 | 27-IV-2011 | Agudo 1 | yes | yes | III | A | PX447962 | PX505133 | PX505208 |
| AA1_clav_Car8 | CAR | CAR5 | *A. clavatus* | 36.69694444 | -3.459166667 | 13 | 27-IV-2011 | Agudo 1 | yes | no | III | na | PX447964 | na | na |
| AA47_clav_Khe1_Oua9 | OUA | OUA | *A. clavatus* | 32.71719444 | -5.799027778 | 762 | 5-VI-2012 | Agudo 47 | yes | yes | I | I | PX447966 | PX505136 | PX505211 |
| IA2016_clav_164 | MUR | MUR1 | *A. clavatus* | 37.604 | -1.136 | 7 | 26-VI-2009 | Álvarez 2016 | yes | no | III | na | PX448014 | na | na |
| IA2016_clav_165 | MUR | MUR2 | *A. clavatus* | 37.604 | -1.136 | 7 | 26-VI-2009 | Álvarez 2016 | yes | yes | III | A | PX448017 | PX505105 | PX505180 |
| IA2016_clav_166 | MUR | MUR3 | *A. clavatus* | 37.604 | -1.136 | 7 | 26-VI-2009 | Álvarez 2016 | yes | yes | III | A | PX448019 | PX505106 | PX505181 |
| IA2045_clav_193 | CEL | CEL1 | *A. clavatus* | 41.64644444 | 2.421388889 | NA | 28-VI-2009 | Álvarez 2045 | yes | no | III | na | PX448009 | na | na |
| IA2045_clav_194 | CEL | CEL2 | *A. clavatus* | 41.64644444 | 2.421388889 | NA | 28-VI-2009 | Álvarez 2045 | yes | no | III | na | PX448010 | na | na |
| IA2050_clav_Cam | CAM | CAM | *A. clavatus* | 42.12422222 | 2.956527778 | 100 | 29-VI-2009 | Álvarez 2050 | yes | yes | III | A | PX447973 | PX505147 | PX505222 |
| IA2069_clav_1356 | VAL | VAL1 | *A. clavatus* | 42.20222222 | -1.645277778 | 250 | 18-VIII-2009 | Álvarez 2069 | yes | yes | III | C | PX448008 | PX505119 | PX505194 |
| IA2069_clav_1357 | VAL | VAL2 | *A. clavatus* | 42.20222222 | -1.645277778 | 250 | 18-VIII-2009 | Álvarez 2069 | yes | yes | III | A | PX448016 | PX505120 | PX505195 |
| IA2069_clav_1358 | VAL | VAL3 | *A. clavatus* | 42.20222222 | -1.645277778 | 250 | 18-VIII-2009 | Álvarez 2069 | yes | yes | III | A | PX448013 | PX505121 | PX505196 |
| IA2073_clav_Fro | FRO | FRO | *A. clavatus* | 42.26770833 | -4.405341667 | 780 | 29-VIII-2009 | Álvarez 2073 | yes | yes | III | H | PX447976 | PX505150 | PX505225 |
| IA2123_clav_TOR | TOR | TOR | *A. clavatus* | 36.96288889 | -4.513555556 | 947 | 28-III-2011 | Álvarez 2123 | yes | yes | III | G | PX447984 | PX505159 | PX505234 |
| IA2135_clav_Sal5 | SAL | SAL1 | *A. clavatus* | 36.72502778 | -3.580666667 | 0 | 30-III-2011 | Álvarez 2135 | yes | yes | III | A | PX447985 | PX505161 | PX505236 |
| IA2135_clav_Sal7 | SAL | SAL2 | *A. clavatus* | 36.72502778 | -3.580666667 | 0 | 30-III-2011 | Álvarez 2135 | no | yes | na | A | na | PX505160 | PX505235 |
| IA2173_clav_1428 | MIR | MIR1 | *A. clavatus* | 40.792925 | -3.733847222 | 906 | 22-X-2011 | Álvarez 2173 | yes | yes | III | A | PX448015 | PX505122 | PX505197 |
| IA2173_clav_1429 | MIR | MIR2 | *A. clavatus* | 40.792925 | -3.733847222 | 906 | 22-X-2011 | Álvarez 2173 | yes | yes | III | A | PX448018 | PX505123 | PX505198 |
| IA2173_clav_1430 | MIR | MIR3 | *A. clavatus* | 40.792925 | -3.733847222 | 906 | 22-X-2011 | Álvarez 2173 | yes | yes | III | A | PX448021 | PX505124 | PX505199 |
| LM4434_clav_Alt5 | ALT | ALT1 | *A. clavatus* | 38.60517222 | -0.049436111 | 25 | 24-V-2008 | Medina 4434 | yes | yes | III | A | PX447993 | PX505170 | PX505245 |
| LM5110_clav_Ita | ITA | ITA | *A. clavatus* | 40.33330556 | 9.531527778 | 175 | 28-VI-2010 | Medina 5110 | yes | yes | III | A | PX447999 | PX505174 | PX505249 |
| IA2333_clav_1588 | RIO | RIO1 | *A. clavatus* | 38.50457222 | -2.448027778 | 1135 | 9-VIII-2013 | Álvarez 2333 | yes | yes | III | A | PX448020 | PX505128 | PX505203 |
| IA2333_clav_1589 | RIO | RIO2 | *A. clavatus* | 38.50457222 | -2.448027778 | 1135 | 9-VIII-2013 | Álvarez 2333 | yes | yes | III | A | PX448036 | PX505129 | PX505204 |
| IA2333_clav_1590 | RIO | RIO3 | *A. clavatus* | 38.50457222 | -2.448027778 | 1135 | 9-VIII-2013 | Álvarez 2333 | no | yes | na | A | na | PX505130 | PX505205 |
| AQ3505_homo_Ask | ASK | ASK | *A. homogamos* | 30.73413889 | -7.797555556 | 1943 | 9-VI-2009 | Quintanar 3505 | yes | yes | I | K | PX447967 | PX505137 | PX505212 |
| IA2115_homo_Asn6 | ASN | ASN1 | *A. homogamos* | 31.25111111 | -7.977777778 | 1160 | 24-V-2010 | Álvarez 2115 | yes | yes | I | K | PX447982 | PX505157 | PX505232 |
| IA2115_homo_Asn8 | ASN | ASN2 | *A. homogamos* | 31.25111111 | -7.977777778 | 1160 | 24-V-2010 | Álvarez 2115 | yes | yes | I | K | PX447981 | PX505156 | PX505231 |
| IA2115_homo_Asn9 | ASN | ASN3 | *A. homogamos* | 31.25111111 | -7.977777778 | 1160 | 24-V-2010 | Álvarez 2115 | yes | yes | I | K | PX447983 | PX505158 | PX505233 |
| RG1275_homo_Imo | IMO | IMO1 | *A. homogamos* | 31.33194444 | -7.408888889 | 2224 | 13-VI-2009 | Gonzalo 1275 | yes | yes | I | L | PX448000 | PX505179 | PX505254 |
| RG1275_homo_Imo6 | IMO | IMO2 | *A. homogamos* | 31.33194444 | -7.408888889 | 2224 | 13-VI-2009 | Gonzalo 1275 | yes | no | I | na | PX448001 | na | na |
| IA2339_line_Mob | MOB | MOB | *A. linearilobus* | 35.76207778 | -0.829633333 | 4 | 23-V-2016 | Álvarez 2339 | yes | yes | II | B | PX447989 | PX505166 | PX505241 |
| IA2345_line_Mac | MAC | MAC | *A. linearilobus* | 35.789625 | -0.152733333 | 2 | 25-V-2016 | Álvarez 2345 | yes | yes | II | I | PX447990 | PX505167 | PX505242 |
| AA33_maroc_Mar | MAR | MAR | *A. maroccanus* | 31.89194444 | -7.439722222 | 570 | 4-VI-2012 | Agudo 33 | yes | yes | I | M | PX447965 | PX505135 | PX505210 |
| IA2097_maroc_Tni | TNI | TNI | *A. maroccanus* | 31.63212778 | -8.252963889 | 380 | 21-V-2010 | Álvarez 2097 | yes | yes | I | J | PX447977 | PX505151 | PX505226 |
| IA2099_maroc | SID | SID | *A. maroccanus* | 31.58801667 | -9.071533333 | 373 | 21-V-2010 | Álvarez 2099 | yes | yes | I | N | PX448004 | PX505152 | PX505227 |
| CA16194_mon | GAB | GAB | *A. monanthos* | 33.88638889 | 10.01838889 | 50 | 23-III-2009 | Aedo 16194 | yes | yes | IV | E | PX448006 | PX505139 | PX505214 |
| CA16233_mon | MAT | MAT | *A. monanthos* | 33.7425 | 10.01555556 | 100 | 23-III-2009 | Aedo 16233 | yes | yes | IV | F | PX448005 | PX505140 | PX505215 |
| CA16310_mon_Zar | ZER | ZER | *A. monanthos* | 33.76034167 | 10.26638889 | 20 | 24-III-2009 | Aedo 16310 | yes | yes | IV | F | PX447968 | PX505141 | PX505216 |
| AQ3531_pyr | ANS | ANS | *A. pyrethrum* | 30.74372222 | -7.706888889 | 2337 | 10-VI-2009 | Quintanar 3531 | yes | yes | I | A | PX448003 | PX505138 | PX505213 |
| IA2334_pyr | PEN | PEN | *A. pyrethrum* | 38.64027778 | -2.378127778 | 1276 | 9-VIII-2013 | Álvarez 2334 | yes | yes | I | A | PX448002 | PX505165 | PX505240 |
| IA2105_radi_coro_Ess | ESS | ESS | *A. radiatus* ssp. *coronatus* | 31.53376389 | -9.743841667 | 5 | 22-V-2010 | Álvarez 2105 | yes | yes | V | D | PX447978 | PX505153 | PX505228 |
| IA2107_radi_coro_Aga | AGA | AGA | *A. radiatus* ssp. *coronatus* | 30.77224167 | -9.803358333 | 286 | 22-V-2010 | Álvarez 2107 | yes | yes | V | J | PX447979 | PX505154 | PX505229 |
| IA2108_radi_coro_Tam | TAM | TAM | *A. radiatus* ssp. *coronatus* | 30.65144722 | -9.886669444 | 4 | 22-V-2010 | Álvarez 2108 | yes | yes | V | J | PX447980 | PX505155 | PX505230 |
| IA2138_radi_radi_Ent | ENT | ENT | *A. radiatus* ssp. *radiatus* | 36.55180556 | -4.696472222 | 35 | 31-III-2011 | Álvarez 2138 | yes | yes | V | A | PX447986 | PX505162 | PX505237 |
| IA2177_radi_radi_Bur | BUR | BUR | *A. radiatus* ssp. *radiatus* | 39.095 | -6.014166667 | 261 | 9-IV-2012 | Álvarez 2177 | yes | yes | V | J | PX447987 | PX505163 | PX505238 |
| IA2180_radi_radi_Che | CHE | CHE | *A. radiatus* ssp. *radiatus* | 35.33775 | -5.362916667 | 640 | 10-IV-2012 | Álvarez 2180 | yes | yes | V | A | PX447988 | PX505164 | PX505239 |
| IA2024_val_Ben | BEN | BEN | *A. valentinus* | 38.71813889 | 0.061944444 | 226 | 27-VI-2009 | Álvarez 2024 | yes | yes | III | A | PX447969 | PX505143 | PX505218 |
| IA2025_val_Xat | XAT | XAT | *A. valentinus* | 38.99588889 | -0.503888889 | 95 | 27-VI-2009 | Álvarez 2025 | yes | yes | II | A | PX447970 | PX505144 | PX505219 |
| IA2030_val_176 | CNV | CNV1 | *A. valentinus* | 39.85722222 | -0.466055556 | 321 | 27-VI-2009 | Álvarez 2030 | yes | yes | II | A | PX448032 | PX505107 | PX505182 |
| IA2030_val_177 | CNV | CNV2 | *A. valentinus* | 39.85722222 | -0.466055556 | 321 | 27-VI-2009 | Álvarez 2030 | yes | yes | II | A | PX448031 | PX505108 | PX505183 |
| IA2032_val_Vin | VIN | VIN | *A. valentinus* | 40.48183333 | 0.47225 | 8 | 27-VI-2009 | Álvarez 2032 | yes | yes | II | H | PX447971 | PX505145 | PX505220 |
| IA2038_val_Alm | ALM | ALM | *A. valentinus* | 40.93513889 | 0.856777778 | 1 | 28-VI-2009 | Álvarez 2038 | yes | yes | II | H | PX447972 | PX505146 | PX505221 |
| IA2041_val_190 | VIL | VIL1 | *A. valentinus* | 41.221 | 1.676055556 | 43 | 28-VI-2009 | Álvarez 2041 | yes | yes | II | A | PX448034 | PX505109 | PX505184 |
| IA2041_val_191 | VIL | VIL2 | *A. valentinus* | 41.221 | 1.676055556 | 43 | 28-VI-2009 | Álvarez 2041 | yes | yes | II | A | PX448033 | PX505110 | PX505185 |
| IA2059_val_Cas | CAS | CAS | *A. valentinus* | 42.26311111 | 3.129305556 | 0 | 29-VI-2009 | Álvarez 2059 | yes | yes | II | O | PX447974 | PX505148 | PX505223 |
| IA2065_val_Elc | ELC | ELC | *A. valentinus* | 42.52519 | -2.622706 | 485 | 17-VIII-2009 | Álvarez 2065 | yes | yes | III | C | PX447975 | PX505149 | PX505224 |
| IA2067_val_1332 | AUT | AUT1 | *A. valentinus* | 42.21305556 | -2.005833333 | 450 | 17-VIII-2009 | Álvarez 2067 | yes | yes | II | A | PX448023 | PX505117 | PX505192 |
| IA2067_val_1334 | AUT | AUT2 | *A. valentinus* | 42.21305556 | -2.005833333 | 450 | 17-VIII-2009 | Álvarez 2067 | yes | yes | II | A | PX448029 | PX505118 | PX505193 |
| IA2137_val_1546 | IZN | IZN1 | *A. valentinus* | 36.77638889 | -4.179222222 | 285 | 30-III-2011 | Álvarez 2137 | yes | yes | III | H | PX448012 | PX505125 | PX505200 |
| IA2137_val_1547 | IZN | IZN2 | *A. valentinus* | 36.77638889 | -4.179222222 | 285 | 30-III-2011 | Álvarez 2137 | yes | yes | II | H | PX448035 | PX505126 | PX505201 |
| IA2137_val_1548 | IZN | IZN3 | *A. valentinus* | 36.77638889 | -4.179222222 | 285 | 30-III-2011 | Álvarez 2137 | yes | yes | II | H | PX448022 | PX505127 | PX505202 |
| IA2182_val_1007 | ALH | ALH1 | *A. valentinus* | 35.24741667 | -3.92447222 | 5 | 11-IV-2012 | Álvarez 2182 | no | yes | na | A | na | PX505115 | PX505190 |
| IA2182_val_1008 | ALH | ALH2 | *A. valentinus* | 35.24741667 | -3.92447222 | 5 | 11-IV-2012 | Álvarez 2182 | yes | yes | II | H | PX448030 | PX505116 | PX505191 |
| IA2182_val_1009 | ALH | ALH3 | *A. valentinus* | 35.24741667 | -3.92447222 | 5 | 11-IV-2012 | Álvarez 2182 | yes | no | II | na | PX448027 | na | na |
| IA2194_val_979 | NAD | NAD1 | *A. valentinus* | 35.17236111 | -2.9338333 | 25 | 12-IV-2012 | Álvarez 2194 | yes | yes | II | A | PX448028 | PX505112 | PX505187 |
| IA2194_val_980 | NAD | NAD2 | *A. valentinus* | 35.17236111 | -2.9338333 | 25 | 12-IV-2012 | Álvarez 2194 | no | yes | na | H | na | PX505113 | PX505188 |
| IA2194_val_981 | NAD | NAD3 | *A. valentinus* | 35.17236111 | -2.9338333 | 25 | 12-IV-2012 | Álvarez 2194 | yes | yes | II | P | PX448024 | PX505114 | PX505189 |
| IA2204_val_968 | OUJ | OUJ1 | *A. valentinus* | 34.7605278 | -1.93802778 | 484 | 12-IV-2012 | Álvarez 2204 | yes | yes | II | A | PX448026 | PX505111 | PX505186 |
| IA2204_val_969 | OUJ | OUJ2 | *A. valentinus* | 34.7605278 | -1.93802778 | 484 | 12-IV-2012 | Álvarez 2204 | yes | no | II | na | PX448025 | na | na |
| IA2336_val | ELA | ELA | *A. valentinus* | 35.70749 | -0.78483 | 128 | 23-V-2016 | Álvarez 2336 | yes | yes | III | A | PX448011 | PX505131 | PX505206 |
| IA2342_val_2 | LAN | LAN | *A. valentinus* | 35.70562 | -0.89241 | 100 | 24-V-2016 | Álvarez 2342 | yes | yes | III | H | PX448007 | PX505142 | PX505217 |
| LM4435_val_Alt10 | ALT | ALT2 | *A. valentinus* | 38.60517222 | -0.049436111 | 25 | 24-V-2008 | Medina 4435 | yes | yes | III | H | PX447994 | PX505171 | PX505246 |
| LM4435_val_Alt11 | ALT | ALT3 | *A. valentinus* | 38.60517222 | -0.049436111 | 25 | 24-V-2008 | Medina 4435 | yes | yes | III | H | PX447996 | PX505175 | PX505250 |
| LM4435_val_Alt5 | ALT | ALT4 | *A. valentinus* | 38.60517222 | -0.049436111 | 25 | 24-V-2008 | Medina 4435 | yes | yes | III | H | PX447997 | PX505176 | PX505251 |
| LM4435_val_Alt6 | ALT | ALT5 | *A. valentinus* | 38.60517222 | -0.049436111 | 25 | 24-V-2008 | Medina 4435 | yes | yes | III | H | PX447995 | PX505172 | PX505247 |
| LM4435_val_Alt9 | ALT | ALT6 | *A. valentinus* | 38.60517222 | -0.049436111 | 25 | 24-V-2008 | Medina 4435 | yes | yes | III | H | PX447998 | PX505173 | PX505248 |
| OH_689_val | COL | COL2 | *A. valentinus* | 41.40638889 | 2.101666667 | 352 | 30-V-2021 | Hidalgo 689 | yes | yes | III | A | PX447958 | PX505177 | PX505252 |
| OH_690_val | COL | COL3 | *A. valentinus* | 41.40638889 | 2.101666667 | 352 | 30-V-2021 | Hidalgo 690 | yes | yes | III | A | PX447959 | PX505178 | PX505253 |
| OH_687_val | COL | COL1 | *A. valentinus* x *A. clavatus* | 41.40638889 | 2.101666667 | 352 | 30-V-2021 | Hidalgo 687 | yes | no | III | na | PX447957 | na | na |
| IA2348_helio_Mou | MOU | MOU | *H. atlantica* | 31.06241389 | -7.937888889 | 3206 | 13-IX-2016 | Álvarez 2348 | yes | yes | Heliocauta | Z | PX447991 | PX505168 | PX505243 |
| IA2350_helio_Tbk | TBK | TBK | *H. atlantica* | 31.06075556 | -7.928297222 | 3721 | 14-IX-2016 | Álvarez 2350 | yes | yes | Heliocauta | Z | PX447992 | PX505169 | PX505244 |
