# Supplementary material for: Inside daisies’ evolutionary tangle: new insights into the hybridogenic genus Anacyclus (Asteraceae, Anthemideae)
Source: PeerJ. 2026 Aug 27;14:e21638. doi: 10.7717/peerj.21638 (PMC13528631; doi:10.7717/peerj.21638)
Supplement: Supplementary file 9 [file peerj-14-21638-s012.docx]

| Taxon | SRA Experiment | Plastome length | Average read coverage | % of N | Plastome Genbank code | 35S rDNA cistron length | 35S rDNA Genbank code |
| --- | --- | --- | --- | --- | --- | --- | --- |
| *Anacyclus clavatus* (Desf.) Pers. | SRX6579297 | 150,131 bp | 48.7 | 0.31 | PV296228 | 5821 bp | PZ437186 |
| *Anacyclus homogamos* (Maire) Humphries | SRX6579295 | 151,266 bp | 52.9 | 0.04 | PV339529 | 5820 bp | PZ437189 |
| *Anacyclus linearilobus* Boiss. & Reuter | SRX6579301 | 151,479 bp | 50.9 | 0.21 | PV339530 | 5814 bp | PZ437184 |
| *Anacyclus maroccanus* (Ball) Ball | SRX6579301 | 150,674 bp | 96.6 | 0.04 | PV339532 | 5820 bp | PZ437190 |
| *Anacyclus monanthos* (L.) Thell. | SRX6579303 | 149,961 bp | 68.2 | 0.19 | PV339526 | 5820 bp | PZ437188 |
| *Anacyclus pyrethrum* (L.) Link | SRX6579293 | 150,164 bp | 59.1 | 0.06 | PV339527 | 5820 bp | PZ437191 |
| *Anacyclus radiatus* Loisel. | SRX6579291 | 151,204 bp | 101.9 | 0.02 | PV339528 | 5821 bp | PZ437187 |
| *Anacyclus valentinus* L. | SRX6579289 | 151,305 bp | 140.6 | 0.02 | PV339525 | 5813 bp | PZ437185 |
| *Heliocauta atlantica* (Litard. & Maire) Humphries | SRX6579287 | 150,060 bp | 406.4 | 0.04 | PV339531 | 5820 bp | PZ437192 |

Table S4. Species information, SRA, plastome and 35S rDNA information for *Anacyclus* spp. and *Heliocauta atlantica*.
