# Supplementary material for: Inside daisies’ evolutionary tangle: new insights into the hybridogenic genus Anacyclus (Asteraceae, Anthemideae)
Source: PeerJ. 2026 Aug 27;14:e21638. doi: 10.7717/peerj.21638 (PMC13528631; doi:10.7717/peerj.21638)
Supplement: Supplementary file 10 [file peerj-14-21638-s013.docx]

**Table S5.** List of genes annotated in the studied plastid genomes of *Anacyclus* spp. and *Heliocauta atlantica*.

| **Gene family** | **Gene** |
| --- | --- |
| Transfer RNA | *trnL-UAG , trnN-GUU , trnRACG,*  *trnA-UGC, trnE-UUC, trnV-GAC,*  *trnL-CAA, trnM-CAU, trnP-UGG,*  *trnW-CCA, trnM-CAU, trnC-ACA*  *trnF-GAA, trnL-UAA, trnT-UGU*  *trnS-GGA, trnM-CAU, trnG-GCC*  *trnS-UGA, trnS-CGA, trnR-UCU*  *trnE-UUC, trnY-GUA, trnD-GUC*  *trnC-GCA, trnS-GCU, trnQ-UUG*  *trnK-UUU, trnH-GUG* |
| Small ribosomal units | *rps*2*, rps*3*, rps*4*, rps*7*, rps*8*, rps*11*, rps*12*, rps*14*, rps*15*, rps*16*, rps*18*, rps*19 |
| Large ribosomal units | *rpl*2*, rpl*14*, rpl*16*, rpl*20*, rpl*22*, rpl*23*, rpl*32*, rpl*33*, rpl*36 |
| RNA polymerase sub-units | *rpo*A*, rpo*B*, rpo*C1*, rpo*C2 |
| Translation initiation facto | *infA* |
| NADH dehydrogenase | *ndh*A*, ndh*B*, ndh*C*, ndh*D*, ndh*E*, ndh*F*, ndh*G*, ndh*H*, ndh*I*, ndh*J*, ndh*K |
| Photosystem I | *pbf*1*, psa*A*, psa*B*, psa*C*, psa*I*, psa*J*, ycf*3*, ycf*4 |
| Photosystem II | *psb*A*, psb*B*, psb*C*, psb*D*, psb*E*, psb*F*, psb*H*, psb*I*, psb*J*, psb*K*, psb*L*, psb*M*, psb*T*, psb*Z |
| Cytochrome b/f complex | *pet*A*, pet*B*, pet*D*, pet*G*, pet*L*, pet*N |
| ATP synthase | *atp*A, *atp*B*, atp*E*, atp*F*, atp*H*, atp*I*, ycf*2 |
| Large subunit of rubisco | *rbcL* |
| Maturase | *matK* |
| Protease | *clpP* |
| Acetyl-CoA-carboxylase sub-unit | *accD* |
| Envelope membrane protein | *cemA* |
| Component of TIC complex | *ycf1* |
| c-Type cytochrome synthesis | *ccsA* |
| Uncharacterized protein | *ycf15* |
| Ribosomal RNA | *rrn*4.5*, rrn*5*, rrn*16*, rrn*23 |
